# Supplementary material for: Global economic impacts of COVID-19 lockdown measures stand out in high-frequency shipping data
Source: PLoS One. 2021 Apr 14;16(4):e0248818. doi: 10.1371/journal.pone.0248818 (PMC8046185; doi:10.1371/journal.pone.0248818)
Supplement: S1 Appendix — (PDF) [file pone.0248818.s001.pdf]

S1 Appendix: Methodology maritime trade estimates

1 Methodology

We briefly describe the methodology to estimate port-level and country-level maritime trade flows based on empirical vessel tracking data. This methodology builds upon Verschuur et al. (2020), who used empirical vessel tracking data to derive port calls on a global scale. We make use of a global database of vessel locations and information that is included in Automatic Identification System (AIS) messages that maritime vessels send out (nowadays a AIS transponder is mandatory for all vessels with a capacity of 300 gross tonnage or more). This data is made available to us through the United Nations Global Working Group on Big Data for Official Statistics. For more information on this data, reference is made to Verschuur et al. (2020). AIS data includes the geospatial location of vessels, including a number of vessel attributes (e.g. length, draft, type, subtype, speed, direction, etc.), every few seconds-minutes. Data is available from 01-2019 to 08-2020.

In short, we develop an algorithm that predicts the type and size (deadweight tonnage) of a vessel based on its dimensions. Using the vessel size and information on draft differences when entering and leaving a port, we can estimate the resulting trade flow (either import or export). We validate this data for five countries that report port-level trade flows (United States, United Kingdom, New Zealand, Brazil and Japan). Additionally, using the classification of the vessel type and the estimated amount that is loaded and unloaded, we create an conversion table that assigns trade to specific sectors based on the vessel type. Combining both levels of information allows us to estimate the volume and value of maritime trade flows on a port, sector and country level. We further validate these trade flows based on an external data source (UN Comtrade).

1.1 Data sources

In order to validate the port-level trade flows, and calibrate the vessel-sector conversion table, we extract monthly port-level data for five countries. Official monthly import and export statistics for the months January-December 2019 are collected from various sources: United States[1], United Kingdom[2], Japan[3], New Zealand[4] and Brazil[5]. These countries are chosen as they are, to the best of our knowledge, the only countries that report monthly port-level import and export values. We match the ports in the respective trade data (ports for United States, United Kingdom and New Zealand,

[1]US Census: https://usatrade.census.gov
[2]UK Revenue and Customs: https://www.uktradeinfo.com
[3]Japan Ministry of Finance: https://www.customs.go.jp/toukei/info/tsdl_e.htm
[4]New Zealand Statistics: http://nzdotstat.stats.govt.nz/wbos/Index.aspx?DataSetCode=TABLECODE7312
[5]Instituto de Pesquisa Econmica Aplicada: http://shiny.ipea.gov.br/comex/

and Custom regions for Japan and Brazil) with the ports in out sample. In total, we have data for 67 ports in the United States, 30 ports in the United Kingdom, 60 ports in Japan, 15 ports in New Zealand and 32 ports in Brazil. However, some remarks should be made. For Brazil and Japan, not all commodities are included in the database, which will result in a likely overestimation of trade flows based on the AIS data. For Japan, this mainly affects exports, as some heavy goods that would contribute much to trade in terms of weight, such as vehicles and heavy machinery, are not included. Moreover, for both Brazil and Japan, the trade flows are associated with custom regions, which are in some cases difficult to match to the correct port (in particular Brazil). Therefore, we have the largest confidence in the comparisons of ports in the United States, New Zealand and the United Kingdom.

1.2 Data preparation

We have manually mapped 1200 port areas across 180 countries to be included in our sub sample of ports. Port areas include the berthing locations and navigation channel to the berths. These ports include all major ports per country and hence cover almost the complete share of global maritime trade as this is mainly concentrated in the large gateway ports. For instance, Trepte and Rice (2014) reported that the largest 20 ports (out of more than 300 ports) in the United States account for more than 80% of the cargo volume in each commodity class.

Using a vessel call algorithm, we extract vessel calls at ports and estimated the turnaround time of vessels in ports. We have implemented several filtering criteria to extract only the port calls that likely contribute to trade. First, we only focus on cargo and tanker vessels. Second, port calls with a turnaround time of less than 5h and more than the 95th percentile (of the port) are truncated, as they are most likely associated with refueling, repair or maintenance. Third, we truncate vessels calls that have a turnaround time of less than 10h and leave the port area at a direction that is within 45 degree of the direction of entering the port area. These port calls are most likely associated with vessels passing a port (e.g. ports alongside a river). This filtering method results in around 3.2 million vessel calls across $\sim$100,000 unique vessels.

1.3 Vessel size and type

To estimate the magnitude of trade, one need information on the carrying capacity of the vessels and the type of vessel. AIS data includes information on the vessel length, width, draught, main type (cargo or tanker) and the subtype (e.g. oil tanker, vehicle carrier, etc.). However, it does not include data on the design draught and carrying capacity, or deadweight tonnage (DWT), of the vessel. Moreover, many data gaps exist, in particular the draft of vessels and the subtype. Here, we used an assimilation method that combines the AIS data and a detailed vessel database with a set of Machine Learning (ML) algorithms to fill in the gaps in the data and estimate the subtype and DWT of the majority of vessels in our sample. The detailed vessel database used here was obtained from a commercial provider (Fleetmon) and includes vessel information for around 38,000 cargo and tanker vessels. First, we predict the DWT of vessels based on vessels dimensions (length, width, draft). Information on the vessel dimensions is assimilated from both data sources. Dimensions included in the vessel database are seen as superior to the AIS data and in case both sources provide data, the vessel database data is taken. To estimate the design draft of the vessel, we search for the maximum draft reported for these vessels within the time frame. We use this design draft in

case the vessel it not included in the vessel database. For data that did not report draft information, we use a RandomForestRegressor algorithm with 500 estimators (robust to changes) to estimate the design draft based on the length and width of a vessel (R^2 = 0.97). Then, we use another RandomForestRegressor algorithm (500 estimators) to estimate the DWT of a vessel based on the vessel dimensions. A RandomForestRegressor is used because it is known that vessel dimensions and DWT have a non-linear relationship (e.g. DTU, 2013). We first split the data between cargo and tanker vessels, as their underlying relationship between dimensions and DWT may differ. We use the vessels included in the vessel database to fit and test the model. Both models have an almost perfect fit (R^2 is 0.99 for both tanker and cargo vessels). The underlying relationship is well captured and the ML algorithm was also able to correct cases of misreporting of the vessel draft in the AIS data (since these are put in manually).

Second, based on the assimilated dataset, we can predict the vessel subtype for those vessels that have no reported subtype. The AIS data includes information on 94 different subtypes. However, not all of these types transport goods. Therefore, we re-group the 94 subtypes into 22 groups of vessel types (see Appendix A for a table) and one group that contains vessel types to be removed (49 vessel subtypes). We now use a RandomForestClassifier algorithm, again using standard configuration, to classify the vessel types based on their dimensions and DWT (again split between cargo and tanker vessels). The prediction rate for cargo vessels equals 90% and for tankers equals 79%, which could be attributed to the limited sample size of tanker vessels (9,651 for tanker versus 21,793 for cargo).

Using both algorithms, we now have a validated dataset with 66,331 unique vessels including their dimensions, carrying capacity and subtype. At last, based on the vessel subtype and DWT, we add the block coefficient of vessels to the database. The block coefficient is ratio of volume displacement of the vessel compared to a rectangular block with the same dimensions (DHI, 2018) and is a important characteristic of the hull of a vessel. We use the block coefficient to predict the payload (or vessel utilization rate) of the vessel based on the reported draft of a vessel (next subsection). Block coefficients for four vessel types (bulk, container, tanker and LNG) and multiple DWT indicators per type are obtained from DHI (2018) and added to the vessel types based on the closest match of DWT per subtype.

1.4 Trade estimate

We use the aforementioned vessel characteristics to estimate the trade flows. To start, some filtering criteria are applied. First, we remove vessels that have a vessel type that does not contribute to trade (not within the 22 categories). Second, for the ports that handle containers, we add a transhipment ratio which will reduce the potentially imported and exported goods that are transported using container vessels (based on data for 70 major transhipment ports). Transhipment covers all goods that are offloaded at a port and then loaded onto another vessel without going through customs. Transhipment accounts for around 28% of global container port throughput with some ports such Singapore, Algeciras (Spain) and Marsaxlokk (Malta) having very high transhipment rates (Notteboom et al., 2019). Excluding transhipments would overestimate trade flows in ports with high transhipment rates. Third, we remove domestic trade flows, as they do not contribute to international trade. We create port-to-port bilateral trade flows based on the port calls. Based on this, we remove

all vessels that travel between ports within the same country and have not visited another country, except for vessels that are classified as container vessels. The latter is to avoid filtering out important trade flows associated with hub and spoke networks of container flows, which represent container vessels that arrive in large hubs, after which containers are moved onto smaller feeder vessels that serve the smaller and more distant ports (Ducruet and Zaidi, 2012, Kavirathna et al., 2018). Fourth, we derive the likely payload (utilisation rate) of the vessel, given that vessels are usually not fully loaded (and it also physically not possible given the weight of crews, fuel, freshwater and supplies). We use the dimensions (length: L, width: W, design draft: d_d), DWT (DWT), block coefficient (Cb) and reported draft (d_r) when entering and leaving a port (for those ports and vessels that have that information) to estimate the payload (μ_v). The block coefficient at reported draft (Cb_r) equals:

$$Cb_r = 1 - \left((1 - Cb_d)\frac{d_r}{d_d}^{1/3}\right) \tag{1}$$

after which the μ_v can be estimated using:

$$\mu_v = \frac{(Cb_r d_r - Cb_d d_d)LW\rho_w + DWT}{DWT} \tag{2}$$

with ρ_w the density in salt water (1029 kg/m^3). For those port calls where draft level is not reported, or where draft when entering and leaving the port is not changing (for which it is unclear whether drafts have been reported or not), we try to backpropagate the draft information by looking at the incoming draft at the next port of call. In case this information is not available, we assign the port-average ingoing and outgoing payload. This payload estimate is of particular importance for ports with large trade imbalance that either have partially full vessels leaving or entering, or have a substantial share of empty containers being carried by container vessels. Lastly, vessels are never completely empty, as they carry ballast water for stability purposes (Jia et al., 2019). Therefore, we assume that if the payload was below 60% when entering and above 60% when leaving when loading (or vice versa when unloading), the vessel can be assumed empty when entering (leaving).

The largest challenge in estimating trade flows is approximating what percentage of the vessel capacity entering will contribute to import and how many goods were exported on the vessel leaving. AIS data does not provide information to what extent cargo was loaded or unloaded when it calls at a port. In the majority of cases, vessels either load or unload goods. Therefore, we estimate the trade flows based on the net unloading (imports) or loading (exports) of vessels, which is estimated based on the draft differences when entering and leaving the port. Estimating trade flows based on the net loading or unloading is also used in previous work (Arslanalp et al., 2019, Cerdeiro et al., 2020). In case there is no difference between the ingoing and outgoing draft (as this is not manually put in), we estimate the ratio of unloading (fraction exports) and loading (fraction imports) based on the imbalance measured at the port. The fraction of imports is found by calculating the net imports over the total trade, for all port calls that do report draft differences:

$$fr_I = \frac{\sum \mu_{v,in} * DWT_{in}}{\sum(\mu_{v,in} * DWT_{in} + \mu_{v,out} * DWT_{out})} \tag{3}$$

Similar for exports:

$$fr_E = \frac{\sum \mu_{v,out} * DWT_{in}}{\sum(\mu_{v,in} * DWT_{in} + \mu_{v,out} * DWT_{out})} \tag{4}$$

In short, we can write:

$$Import = \begin{cases} fr_I * DWT_{in}, & \text{if } d_{in} - d_{out} = 0 \\ \mu_{v,in} * DWT_{in} - \mu_{v,out} * DWT_{out}, & \text{if } d_{in} - d_{out} > 0 \\ 0, & \text{if } d_{in} - d_{out} < 0 \end{cases} \tag{5}$$

$$Export = \begin{cases} fr_E * DWT_{out}, & \text{if } d_{in} - d_{out} = 0 \\ \mu_{v,out} * DWT_{out} - \mu_{v,in} * DWT_{in}, & \text{if } d_{out} - d_{in} > 0 \\ 0, & \text{if } d_{out} - d_{in} < 0 \end{cases} \tag{6}$$

Using this methodology when can partly correct for trade flows in ports with large trade imbalances, as vessels in these ports will be predominantly loaded or unloaded. The accuracy of the method depends on the level of draft reporting in ports, which varies strongly globally. In particular, many Caribbean, Latin American, Northern African, and South-East Asian countries have low draught reporting values. Hence, we expect an error term that is consistent within countries, but varies between countries.

1.5 Sector-level trade and conversion from volume to value

In order to link these imports and exports to economic sectors, we establish a conversion table that describes the probability that a certain vessel types is associated with a particular economic sector. In this way, the goods imported and exported per vessel type can be disaggregated to economic sectors. We extract commodity-level import and export data per country, which are converted to a coherent classification system[6]. We use the 56 economic sector classification system as used in the World Input-Output Tables (see Dietzenbacher et al., 2013), which is based on the 'International Standard Industrial Classification of All Economic Activities' (ISIC). From these 56, we only focus on the first 22 sectors, as these sectors include the majority of commodities that are expected to be transported by maritime transport (Appendix B).

We match the data for the ports and months with the AIS data and end up with two matrices: one that represent the import and export per port for every given month and economic sector, and one that includes AIS-derived import and export disaggregated to vessel types per month. The resulting conversion table needs to be a nxm matrix with n the number of economic sectors and m the number of vessel types. We first normalise both matrices per row, which makes it easier to solve. We decide to make the conversion table only based on data for the United Kingdom, New Zealand, United Kingdom and Japanese imports, given the highest expected accuracy. We end up with a X x 21 matrix (A) with AIS derived trade flows per vessel type and a X x 22 matrix (B) with official trade flows. We solve this by defining a minimization problem using linear programming. We add two constraints to the solution: all elements in the solution are larger or equal than 0, and the sum per row is equal to 1. The latter is to ensure that the cargo load that enters or leaves a port is fully distributed over the associated economic sectors. For instance, if a vessel with type X enters a ports, its cargo load is distributed 20% into sector A, 30% into sector B, 15% into sector C, and 35% into sector D. In matrix form, this reads:

[6] The conversion tables for the classification systems are taken from https://unstats.un.org/unsd/classifications/Econ

$$
\begin{aligned}
\min \quad & |\mathbf{AX} - \mathbf{B}|^2 \\
\text{s.t.} \quad & \mathbf{X}(n,m) \geq 0 \\
& \sum_{i \in n} \mathbf{X}(i,m) = 1
\end{aligned}
\tag{7}
$$

Using this conversion table, we can translate the AIS derived imports and exports per vessel types to imports and exports per economic sector, which can be compared to the custom data. The conversion table fitted is however partially a characterisation of the countries the data is calibrated to. Therefore, we add a correction factor that compares the sector presence in the country of interest to the calibration countries. For instance, it could be that bulk carriers transport grains in agriculture exporting countries, whereas they carry iron ore in mining heavy countries. This correction factor is based on volume imports and exports per HS6 code on a national level derived from 2018 trade data from the BACI database (Gaulier and Zignago, 2010), which we translate from the HS6 classification to the economic sectors used in this study. It can be seen as a ratio of the sector distribution per trade flow ($ratio_{s,i} = \%_{s,i}/\%_{s,base}$) with s the sector, i the country of interest and $base$ the data used for deriving the conversation table.

Additionally, we create a conversion table to translate volume into value, as different sectors have vastly different values of imported and exported goods. To do this, we create a country-specific and sector-specific conversion table that translates sector-specific volume to value (for both import and export). Both conversion tables are constructed using the BACI trade database (Gaulier and Zignago, 2010). We assume that the conversion values for maritime trade is similar as the conversion values for all modes of transport. We end up with sector-specific trade flows on a port-level.

We further aggregate the sector-level data to a 11-sector classification, which will improve the accuracy of the sector estimates. These 11 sectors are included in the Appendix C.

2 Validation

We perform a number of validations: (1) monthly port-level trade flows per country, (2) a sector-level validation on a country-level, and (3) an external validation based on UN Comtrade data for the countries that report maritime trade flows.

2.1 Port-level validation

S1 Figure 1 compares the monthly trade flows estimated using the AIS with the customs data per country. A good agreement is found for all countries. Some observations can be made. First, an overprediction is found in case a country has a large trade imbalance. For instance, the United Kingdom imports 4.3 times more in terms of volume than it exports, hence an overestimation is found for exports. This is because the method cannot correct enough for the large imbalance that exist at a port-level. A similar observation can be made for Brazil, exporting 5.9 times more in terms of volume than it imports, resulting in an overall overprediction for imports. For Japan, the exports are largely overpredicted, but we hypothesize that this can be attributed to the missing exports in the custom

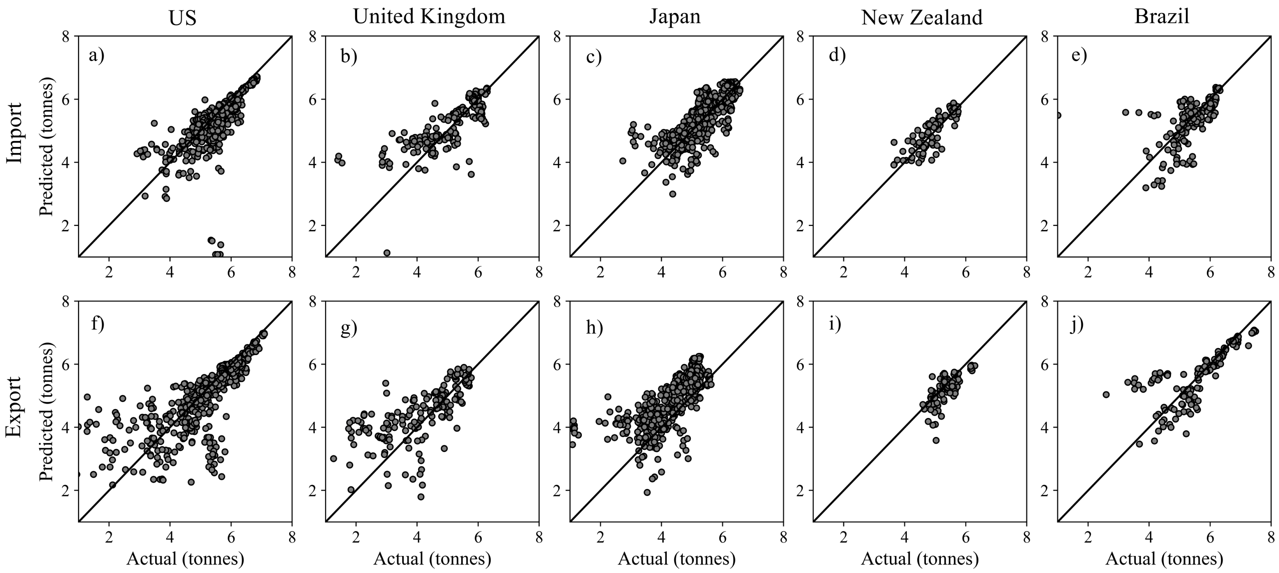

S1 Figure 1: Comparison observed and predicted trade flows on a port level for the US, UK, Japan, New Zealand and Brazil. Note that all estimates are log10 estimates.

data (commodities that do not have a volume-based unit such as vehicles). Second, the prediction for small ports is generally less accurate than the larger ports, which can be observed in the US, the United Kingdom and Brazil. Smaller ports are in general less diversified (Ducruet et al., 2010, 2015), therefore serving only a few specialised industries. Such specialised industries, such as raw materials, usually have a large trade imbalance (e.g. export-orientated for raw materials for export countries and import-orientated for import countries), making it hard to predict trade.

On a port-level, the correlation coefficient ranges from 0.52-0.96, and R^2 values are in the order of 0.32-0.85 (except for Japanese exports). Moreover, we compare the monthly average (aggregated) imports and exports on a country-level. Most trade flows are within a 40% deviation, with only Japan exports having a larger overprediction (4 times), due to the issues with the customs data. Trade flows for the United States are underpredicted, whereas trade flows for United Kingdom (both imports and exports), New Zealand imports and Brazil imports are well-captured.

2.2 Sector-level validation

We also validate the aggregated trade flows per sector per country. The result are shown in S1 Figure 2, showing considerable improvement over the port-level estimates. For this comparison, the correlation coefficients vary between 0.79-0.98 and R^2 values between 0.39-0.89. On a national level, the overprediction of sector-specific trade flows in small ports become negligible, indicating that this method works well on a national scale.

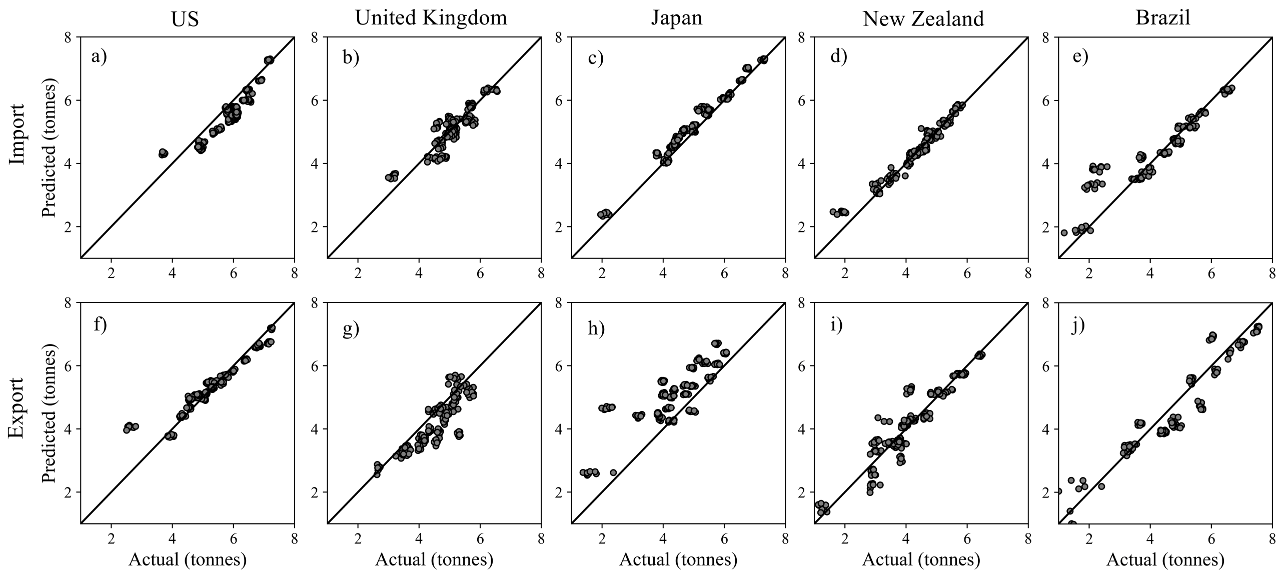

S1 Figure 2: Comparison observed and predicted sector-level trade flows on a country level for the US, UK, Japan, New Zealand and Brazil. Note that all estimates are log10 estimates.

2.3 External validation UN Comtrade

As external validation, we collect monthly mode of transport data from the UN Comtrade database (https://comtrade.un.org) (Jan 2019 - December 2019), which now allows countries to include mode of transport when reporting trade. For 2019, we find 28 countries reporting maritime imports and 27 reporting maritime exports and compare the maritime trade flows in terms of value for these countries. We compare both the aggregate monthly values on a country-level (top S1 Figure 3) as well as the sector-specific trade flows (bottom S1 Figure 3).

The aggregate trade flows (top S1 Figure 3) show a very good fit for most countries. Smaller trade flows are harder to predict, most likely due to a lower coverage of draft reporting (as smaller maritime trade flows are more common in low-income countries with lower reporting frequency) and potentially large trade imbalances (e.g. small islands). The correlations coefficients for imports and exports are 0.84 and 0.86, respectively. Moreover, the sector-specific data shows a similar pattern as observed above, with smaller trade flows and smaller sectors having a larger errors than larger trade flows of more dominant sector. The correlation coefficients are found to be 0.78 for imports and 0.73 for exports.

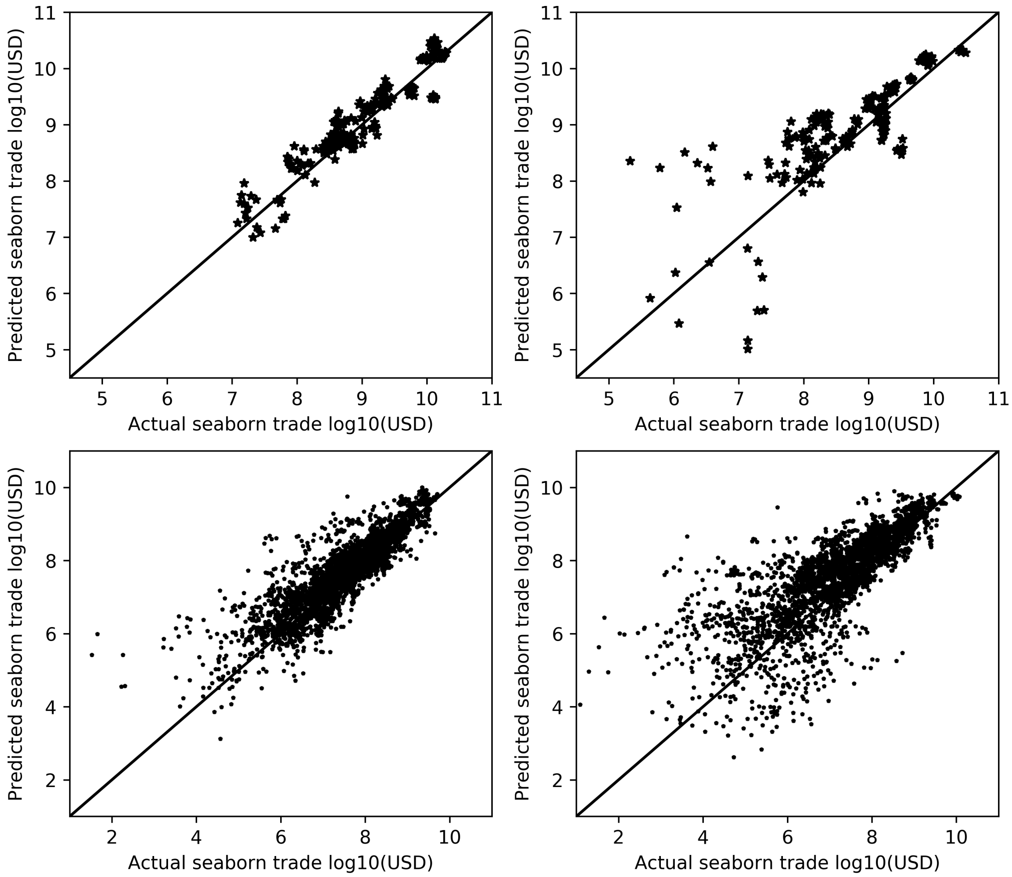

S1 Figure 3: Comparison observed and predicted maritime trade flows on a country level for 28/27 countries reporting mode of transport data for imports/exports. Top left figure: monthly maritime imports on a country-level. Top right figure: monthly maritime exports on a country-level. Bottom left figure: monthly maritime imports on a sector and country-level. Bottom right figure: monthly maritime exports on a sector and country-level. Note that all estimates are log10 estimates.

References

Arslanalp, S., M. Marini, and P. Tumbarello (2019). Big Data on Vessel Traffic : Nowcasting Trade Flows in Real Time. Imf working paper wp/19/275, International Monetary Funds.

Cerdeiro, D. A., A. Komaromi, Y. Liu, and M. Saeed (2020). World Seaborne Trade in Real Time : A Proof of Concept for Building AIS-based Nowcasts from Scratch. IMF Working paper WP/20/XX, International Monetary Fund.

DHI (2018). Dhi vessel hulls catalogue: data sheets for different vessels in mike 21 mooring analysis. Technical report, DHI Group, Denmark.

Dietzenbacher, E., B. Los, R. Stehrer, M. Timmer, and G. de Vries (2013). the Construction of World Input-Output Tables in the Wiod Project. *Economic Systems Research 25*(1), 71–98.

DTU (2013). Statistical analysis and determination of regression formulas for main dimensions of container ships based on ihs fairplay data. Project no. 2010-56,emissionsbeslutningsstttesystem, work package 2, report no. 03, Technical University of Denmark.

Ducruet, C., H. Itoh, and O. Joly (2015, aug). Ports and the local embedding of commodity flows. *Papers in Regional Science 94*(3), 607–627.

Ducruet, C., H. R. Koster, and D. J. van der Beek (2010). Commodity variety and seaport performance. *Regional Studies 44*(9), 1221–1240.

Ducruet, C. and F. Zaidi (2012). Maritime constellations: a complex network approach to shipping and ports. *Maritime Policy and Management 39*(2), 151–168.

Gaulier, G. and S. Zignago (2010, October). Baci: International trade database at the product-level. the 1994-2007 version. Working Papers 2010-23, CEPII.

Jia, H., V. Prakash, and T. Smith (2019). Estimating vessel payloads in bulk shipping using AIS data. *International Journal of Shipping and Transport Logistics 11*(1), 25–40.

Kavirathna, C., T. Kawasaki, S. Hanaoka, and T. Matsuda (2018). Transshipment hub port selection criteria by shipping lines: the case of hub ports around the bay of Bengal. *Journal of Shipping and Trade 3*(1).

Notteboom, T. E., F. Parola, and G. Satta (2019). The relationship between transhipment incidence and throughput volatility in North European and Mediterranean container ports. *Journal of Transport Geography 74*, 371–381.

Trepte, K. and J. B. Rice (2014). An initial exploration of port capacity bottlenecks in the USA port system and the implications on resilience. *International Journal of Shipping and Transport Logistics 6*(3), 339–355.

Verschuur, J., E. Koks, and J. Hall (2020). Port disruptions due to natural disasters: Insights into port and logistics resilience. *Transportation Research Part D: Transport and Environment 85*, 102393.

Appendix A: Vessel types

Group	Vessel types
Bitumen Tanker	Bitumen Tanker, Asphalt Bitumen Tanker
Cement Carrier	Cement Carrier, Aggregates Carrier, Aggregates Carrier, Limestone Carrier, Powder Carrier
Chemical Products	Chemical Oil Products Tanker, Caprolactam Tanker, Urea Carrier
Chemical Tanker	Chemical Tanker
Coal Oil Mixture Tanker	Coal Oil Mixture Tanker
Container	Container Ship, Barge Carrier, Container Ro Ro Cargo Ship
Dry Bulk	Bulk Carrier, Self Discharging Bulk Carrier, Bulk Oil Carrier
Forest	Wood Chips Carrier
General Cargo	General Cargo Ship, Trans Shipment Vessel, Deck Cargo Ship, Palletized Cargo Ship<br>Heavy Load Carrier, Passenger General Cargo Ship
Animal products	Livestock Carrier, Live Fish Carrier, Fish Carrier, Fish Factory Ship
LPG/LNG	Lpg Tanker, Lng Tanker, Fsru, Combination Gas Tanker Lng Lpg, Co2 Tanker, Fpso
Molasses Tanker	Molasses Tanker
Oil And Chemical Tanker	Oil And Chemical Tanker
Oil Products	Oil Products Tanker
Oil Tanker	Tank Barge, Crude Oil Tanker
Ore Carrier	Ore Carrier, Ore Oil Carrier
Other Tanker	Other Tanker
Reefer	Refrigerated Cargo Ship, Fruit Juice Tanker
Refined Sugar Carrier	Refined Sugar Carrier
Ro Ro Cargo Ship	Ro Ro Cargo Ship
Vegetable Oil Tanker	Vegetable Oil Tanker, Edible Oil Tanker
Vehicles Carrier	Vehicles Carrier
Vessel types to be removed	Yacht, Patrol Vessel, Tug, Service Ship, Pusher Tug, Passenger Ship, Offshore Tug Supply Ship<br>Sailing Vessel, Dredger, Research Vessel, Hopper Dredger, Work Repair Vessel, Split Hopper Barge<br>Fishing Vessel, Offshore Vessel, Crewboat, Buoy Lighthouse Vessel, Hopper Barge, Cable Layer<br>Offshore Support Vessel, Bunkering Tanker, Fishing Support Vessel, Pipe Layer, Drilling Ship, Pilot Vessel<br>Offshore Supply Ship, Pollution Control Vessel, Offshore Supply Ship, Salvage Ship, Cruise Ship<br>Crane Ship, Water Tanker, Waste Disposal Vessel, Utility Vessel, Landing Craft, Well Stimulation Vessel<br>Search And Rescue Vessel, Standby Safety Vessel, Training Ship, Offshore Processing Ship, Passenger Ro Ro Cargo Ship

S1 Table 1: Overview of the vessel groups and the vessel subcategories that are included per group.

Appendix B: sectors for conversion

Sector number	Description
1	Crop and animal production, hunting and related service activities
2	Forestry and logging
3	Fishing and aquaculture
4	Mining and quarrying
5	Manufacture of food products, beverages and tobacco products
6	Manufacture of textiles, wearing apparel and leather products
7	Manufacture of wood and of products of wood and cork, except furniture; manufacture of articles of straw and plaiting materials
8	Manufacture of paper and paper products
9	Printing and reproduction of recorded media
10	Manufacture of coke and refined petroleum products
11	Manufacture of chemicals and chemical products
12	Manufacture of basic pharmaceutical products and pharmaceutical preparations
13	Manufacture of rubber and plastic products
14	Manufacture of other non-metallic mineral products
15	Manufacture of basic metals
16	Manufacture of fabricated metal products, except machinery and equipment
17	Manufacture of computer, electronic and optical products
18	Manufacture of electrical equipment
19	Manufacture of machinery and equipment n.e.c.
20	Manufacture of motor vehicles, trailers and semi-trailers
21	Manufacture of other transport equipment
22	Manufacture of furniture; other manufacturing

S1 Table 2: Overview of economic sectors in accordance with the World Input-Output Tables (Dietzenbacher et al., 2013).

Appendix C: final sector classification

Sector number	Description
1	Agriculture
2	Fishing
3	Mining and Quarrying
4	Food & Beverages
5	Textiles and Wearing Apparel
6	Wood and Paper
7	Petroleum, Chemical and Non-Metallic Mineral Products
8	Metal Products
9	Electrical and Machinery
10	Transport Equipment
11	Other Manufacturing

S1 Table 3: Overview of economic sectors used in this work.
