# Supplementary material for: Global economic impacts of COVID-19 lockdown measures stand out in high-frequency shipping data
Source: PLoS One. 2021 Apr 14;16(4):e0248818. doi: 10.1371/journal.pone.0248818 (PMC8046185; doi:10.1371/journal.pone.0248818)
Supplement: S2 Appendix — (PDF) [file pone.0248818.s002.pdf]

S2 Appendix: econometric model

Model description and formulation

To estimate the effect of various NPI on daily maritime exports, we apply reduced-form econometrics [1] using a fixed effects panel regression model. A fixed effects model, instead of a random effects model, was chosen after performing the Hausman test [2] (which compares a fixed effects and random effects model) for both the regression model using the composite index and the individual NPI. For both models, we had to reject the null hypothesis (at p >0.05 and p>0.1), indicating that there is strong evidence to suggest that country-specific variables that are correlated with included explanatory variables have been omitted. Hence, adopting a random effects model would result in biased estimates. The fact that a larger number of country-specific variables (beyond what we control for) are important is also shown in Furceri et al. [3], who concluded that health-related factors such as death per capita, cases per capita, health preparedness and stringency of NPI only explain a small fraction of the variance in economic output losses across countries. Additionally, factors such as GDP/capita, tourism share, social fractionalization, liberalized credit markets, pre-crisis growth and whether a regime is democratic or not have an influence on the extent of output losses among countries.

We follow a similar implementation of the model as done in Deb et al. [4], although we control more specifically for the potential supply and demand shock. For every country (i), we derive country-wide daily (t) indicators of the change in maritime exports ($\Delta E_{i,t}$) by aggregating the daily time series of estimated export data on a port-level. We use exports as an indicator of industry output as it better reflects the status of the economy compared to changes in imports, which are likely influenced by supply shortages (due to reduced output in trade-dependent countries) and reduced demand for products (due to imposed lockdowns). We detrend (using a linear regression) the 2019 time series in order to filter out a structural increase or decrease in maritime exports. The trend is only removed if a statistically significant signal is observed ($p < 0.05$). We smooth the data using a 10-day moving average, which is necessary to reduce the noise for countries with highly variable daily export estimates (e.g. smaller economies). Smoothing the (daily) time series of high-frequency data is a common way to improve the signal the noise ratio and remove unwanted cycles. For instance, we find a weekly cycle in exports in some countries (slightly higher or lower trade on weekend days), which we want to filter out. Moving averages of 5-14 days have been used in order studies that use high-frequency datasets [4–7]. We do note that smoothing the time series introduces autocorrelation in our error term, but because the fixed-effect model clusters standard errors at the country-level, this will not influence our model fit (as this makes it robust against autocorrelation). We compare the 2020 time series (Jan – Aug) to the time series for 2019 and estimate the percentage change deviation from the average daily exports (based on 2019 daily data). We remove countries where the daily exports are zero for at least one day, as this will likely bias the results (e.g. some

small islands only export products on certain days depending on the arrival of maritime vessels).

For every country, we retrieve information about the government policy responses from the Oxford COVID-19 Government Response Tracker (OxCGRT) [8]. We obtained information on nine NPI that potentially affect business operations: C1 - School closing; C2 - Workplace closing; C3 - cancel public events; C4 - Restrictions on gatherings; C5 - Close public transport; C6 - Stay at home; C7 - Restrictions on internal movement; C8 - International travel controls; H2 - Testing policy. These measures all have different ordinal scales depending on the different levels of responses (e.g. restrictions internal movements has two levels, whereas international travels restrictions has four levels). We normalise all policies to a 0 to 1 range, with 0 implying no measure implemented and 1 referring to the maximum severity of the measure. This provides us with a set of daily policy responses (p) per country ($c_{p,i,t}$). We also derive an overall Stringency metric that is the sum of all policies (C1-C8) normalised to a 0 to 1 scale ($S_{i,t}$).

We control for several factors ($X_{i,t}$), including (1) the daily number of confirmed cases, (2) the supply-shock, and (3) the demand-shock, since these factor can cause variations in the export change across countries. First, the daily number of confirmed cases is an indicator of the severity of the health crisis and was found to be important to explain differences in output losses across countries [3]. Daily values of the number of cases are obtained from OxCGRT [8], which we divide over a country's population in order to estimate the fraction of the population that is positively tested on a particular day. Second, we control for the potential effect of a supply shock that affects a country's ability to export products, irrespective of having NPI implemented. The supply-shock was particularly prevalent in the beginning of the pandemic when initially unaffected countries had to reduce exports because some important imported goods did not enter their supply-chains. Cerdeiro and Komaromi [7] used a similar high-frequency maritime trade dataset (although with some differences in methodology) in order to test whether the occurrence of supply spillovers from lockdowns in trade-dependent countries was found in reality. Using a shift-share identification strategy they showed how supply shocks were transmitted through international maritime trade, although this evidence was only strong in the early stages of the pandemic. To capture this supply-side effects, and how it might propagate downstream to changes in exports, we derive the daily, sector-specific, changes in imports that are used to produce export products. To do this, we create a sector-specific vertical specialisation coefficient per country, as proposed by Hummels et al. [9], using the 2015 EORA multi-regional input-output tables [10]. This coefficient reflect the dollar value increase in imports for every dollar increase in exports in a country, including all industry interdependencies. By multiplying this coefficient with the sector-specific daily import data per country, we estimate the daily time series of supply of goods that are used to produce exports. We again derive daily percentage changes in the supply (similar as for the export time series, see above). Third, countries might directly reduce exports when demand in

trade-depending countries changes. For instance, Verschuur et al. [11] showed how iron ore exports in Australia declined when China, the main importing country of Australian iron ore, went into their first lockdown. To capture this potential effect, a daily time series of the demand shock is derived by estimating the weighted average stringency value in trade-dependent countries, assuming that countries that have higher stringency values demand less products. The stringency values are derived from OxCGRT [8] and range between 0 and 100. We extract data from the 2018 (latest year available) BACI harmonized trade database [12] and use this to estimate the daily demand shock by multiplying the stringency value of the importing country with the fraction the bilateral trade flow (between exporting and importing country) contributes to the total exports of exporting country (weight). For every export country, we sum over the total number of trade relationships to end up with a weighted mean stringency value. In this way, we account for the fact that exports can reduce before a country implemented NPI. We hereby assume that a demand shock happens instantaneously without any lag (businesses cancelling orders which directly reduces export in trade-dependent countries). At last, we add a three day lag of the export change ($\Delta E_{i,t-\Delta t}$) itself to the model in order to effectively control for the normal dynamics in daily exports and other endogenous factors that are likely to be serially correlated with daily exports.

In summary, we can express the daily change in exports in 2020 compared to 2019 as:

$$\Delta E_{i,t} = \mu_i + \tau_t + c_{p,i,t} + X_{i,t} + \Delta E_{i,t-\Delta t} + \epsilon_{i,t} \qquad (1)$$

with μ_i the country-specific fixed effect to account for time-invariant country characteristics and τ_t a time fixed effect (either day, week or month) to account for changes in the global economy that affects exports across countries. We also tested implementing the policies as separate dummy variables per ordinal scale, in order to capture non-linear effects, which gives similar results, although it becomes harder to estimate the effect of the individual NPI.
Alternatively, we can replace the individual NPI with the overall Stringency estimate:

$$\Delta E_{i,t} = \mu_i + \tau_t + S_{i,t} + X_{i,t} + \Delta E_{i,t-\Delta t} + \epsilon_{i,t} \qquad (2)$$

The results of these models are included in Table 3 in the main article.

Sensitivity analysis

Throughout the analysis, we made some non-intuitive modelling decisions that might influence the results. Therefore, to improve transparency, we perform a sensitivity analysis to evaluate how changes in the decisions shape the results.

First, we test the number of days used to smooth the daily trade estimates. As mentioned before, previous studies, analysing high-frequency mobility, emissions and maritime transport data, have used values between 5-14 days in their analysis. We compare the 10 day smoothing with a 7 day and a 3 day smoothing (note that with the 3 day smoothing we change the lag of exports to 1 day to not bias the results). Decreasing the smoothing will generally decrease the signal to noise ratio, making it harder to establish the effect of NPI. The results are added to S2 Table 1.

Second, we change the lag of the export change from the original 3 days to a 5 and 7 day value. By increasing the lag in Equation 1 and 2, the effect of the other endogenous factors that control export change becomes less strong, attributing more weight to the NPI. Because it is not clear what value should be adopted (as we do not know explicitly what these factors are), it is important to check the robustness of the results for different assumptions. The results are included in S2 Table 2.

Third, we change the time fixed effects in the model from day to a week or month fixed effects, as this might change to what extent factors in the global economy that influence export across countries are controlled for. Although a day fixed effects might capture most of the dynamics, there is a risk that it might capture too much, since at some points in time, the majority of countries were in lockdown. The comparison is shown in S2 Table 3, and as expected, shows that the effect of the NPI tend to increase if week or month fixed effects are included.

Robustness checks

Moreover, we implement two robustness checks to evaluate the effect of implementing the policies in a lagged manner and to check whether potential multicollinearity between the policies influences the results.

Accounting for the lag structure is important as it has not yet been demonstrated that introducing NPI will immediately affect export change following implementation. Hence, one would expect the estimated coefficient to increase if a lagged response is prevalent, whereas a decrease in the coefficient would indicate the opposite. To evaluate this, we set-up a ‘fixed-lag’ model, similar as done in Hsiang et al. [13], in which we implement the individual policies in a lagged manner ($c_{p,i,t-\Delta tc}$) with Δtc = 0/5/10 days:

$$\Delta E_{i,t} = \mu_i + \tau_t + c_{p,i,t-\Delta tc} + X_{i,t} + \Delta E_{i,t-\Delta t} + \epsilon_{i,t} \quad (3)$$

The results are shown in S2 Table 4.

Additionally, we are concerned that multicollinearity between the policies in Equation 1 might influence the estimated coefficients for the NPI. Multicollinearity arises because governments often impose multiple NPI at the same time after periodically re-

evaluating the effect they have on cases/deaths. Therefore, in Equation 1, we implement the policies one-by-one (c_p is C1 to C8) instead of all-at-the-same-time. Although this obviously introduces omitted variables bias to the model (because we know that policies were introduced simultaneously), it acts as a robustness check to test whether the effects of the NPI is found under both model specifications. The results are included in S2 Table 5.

S2 Table 1: Regression results for the model specifications that include a varying number of days to smooth the trade estimates (10/7/3 days smoothing). *p < 0.1, **p < 0.05, ***p < 0.01

Parameter	10 day Beta	7 day Beta	3 day Beta	10 day Beta	7 day Beta	3 day Beta
Composite				-4.003**	-3.310*	-3.982
C1	-3.968***	-2.802*	-4.880**			
C2	-2.891**	-5.891***	-2.275			
C3	0.012	-0.699	-2.471			
C4	-2.375*	-0.506	2.780			
C5	-3.864***	-2.288*	-3.838*			
C6	3.857**	1.848	-2.962			
C7	2.729**	3.563***	6.033***			
C8	3.347***	4.367***	4.656***			
H2	0.960	-0.236	-5.117*			
Demand	-0.026	-0.002	-0.192***	-0.021	0.000	-0.173***
Cases	-0.179***	-0.1311	-0.196*	-0.167**	-0.124	0.163
Supply	0.068***	0.067***	0.041***	0.068***	0.067***	0.041***
Export lag	0.372***	0.288***	0.100***	0.373***	0.290***	0.100***
R2	0.350	0.241	0.093	0.348	0.254	0.091
R2-adjusted	0.339	0.228	0.075	0.337	0.241	0.073
F-statistic	31.77	18.46	5.01	32.41	19.63	5.04

S2 Table 2: Regression results for the model specifications that include a varying number of days for the lag in the export change itself (3/5/7 days lag). *p < 0.1, **p < 0.05, ***p < 0.01

Parameter	3 day Beta	5 day Beta	7 day Beta	3 day Beta	5 day Beta	7 day Beta
Composite				-4.003**	-4.865***	-5.845***
C1	-3.968***	-4.561***	-5.032***			
C2	-2.891**	-3.703**	-4.165***			
C3	0.012	-0.401	-0.601			
C4	-2.375*	2.300*	-2.236			
C5	-3.864***	-4.277***	-4.668***			
C6	3.857**	4.717***	5.063***			
C7	2.729**	3.029***	3.296***			
C8	3.347***	3.432***	3.127***			
H2	0.960	1.156	1.110			
Demand	-0.026	-0.021	-0.044	-0.021	-0.018	-0.043
Cases	-0.179***	-0.233***	-0.267***	-0.167**	-0.218***	-0.252***
Supply	0.068***	0.081***	0.090***	0.068***	0.081***	0.090***
Export lag	0.372***	0.213***	0.115***	0.373***	0.214***	0.116***
R2	0.350	0.222	0.165	0.348	0.219	0.162
R2-adjusted	0.339	0.208	0.151	0.337	0.206	0.149
F-statistic	31.77	16.76	11.58	32.41	17.01	11.68

S2 Table 3: Regression results for the model specifications that include a different time fixed effects (day/week/month) *p < 0.1, **p < 0.05, ***p < 0.01

Parameter	day<br>Beta	week<br>Beta	month<br>Beta	day<br>Beta	week<br>Beta	month<br>Beta
Composite				-4.003**	-4.282***	-4.450***
C1	-3.968***	-3.916***	-3.917***			
C2	-2.891**	-2.848**	-2.904**			
C3	0.012	-0.253	-0.585			
C4	-2.375*	-2.447**	-2.170*			
C5	-3.864***	-3.849***	-3.728***			
C6	3.857**	3.843**	3.540**			
C7	2.729**	2.805**	2.975***			
C8	3.347***	3.117**	3.242***			
H2	0.960	0.693	0.719			
Demand	-0.026	-0.045	-0.046*	-0.021	-0.040	-0.047*
Cases	-0.179***	-0.165**	-0.158**	-0.167**	-0.151**	-0.144**
Supply	0.068***	0.068***	0.067***	0.068***	0.067***	0.067***
Export lag	0.372***	0.371***	0.371***	0.373***	0.372***	0.372***
R2	0.350	0.345	0.342	0.348	0.343	0.340
R2-adjusted	0.339	0.339	0.337	0.337	0.338	0.336
F-statistic	31.77	63.63	72.64	32.41	66.79	76.92

S2 Table 4: Regression results for the model specifications by introducing a lagged implementation of the NPI (0/5/10 days lag). *p < 0.1, **p < 0.05, ***p < 0.01

Parameter	Base Beta	5 day Beta	10 day Beta	Base Beta	5 day Beta	10 day Beta
Composite				-4.003**	-4.839***	-3.897**
C1	-3.968***	-5.237***	-8.078***			
C2	-2.891**	-1.649	-0.265			
C3	0.012	0.926	-0.303			
C4	-2.375*	-1.814	-1.396			
C5	-3.864***	-3.358***	-2.444**			
C6	3.857**	3.458**	5.025***			
C7	2.729**	1.122	0.253			
C8	3.347***	3.125***	6.051***			
H2	0.960	0.748	1.067			
Demand	-0.026	-0.021	-0.022	-0.021	-0.020	-0.023
Cases	-0.179***	-0.160**	-0.149**	-0.167**	-0.158**	-0.153**
Supply	0.068***	0.068***	0.068***	0.068***	0.067***	0.067***
Export lag	0.372***	0.372***	0.372***	0.373***	0.373***	0.373***
R2	0.350	0.349	0.351	0.348	0.348	0.348
R2-adjusted	0.339	0.338	0.340	0.337	0.337	0.337
F-statistic	31.77	31.73	31.92	32.41	32.42	32.41

S2 Table 5: Regression results for the model specifications in which the NPI are introduced one by one instead of all at the same time. *p < 0.1, **p < 0.05, ***p < 0.01

Parameter	C1 only Beta	C2 only Beta	C3 only Beta	C4 only Beta	C5 only Beta	C6 only Beta	C7 only Beta	C8 only Beta
C1	-3.438***							
C2		-4.096***						
C3			-2.281**					
C4				-3.278***				
C5					-3.823***			
C6						0.407		
C7							0.518	
C8								1.931*
Demand	-0.003	-0.000	-0.011	-0.012	-0.013	-0.018	-0.019	-0.024
Cases	-0.171***	-0.155**	-0.175***	-0.167**	-0.175**	-0.165**	-0.166**	-0.153**
Supply	0.067***	0.066***	0.066***	0.067***	0.066***	0.067***	0.067***	0.067***
Export lag	0.374***	0.374***	0.374***	0.375***	0.374***	0.375***	0.375***	0.375***
R2	0.348	0.348	0.348	0.348	0.348	0.348	0.348	0.348
R2-adjusted	0.337	0.338	0.337	0.337	0.338	0.337	0.337	0.337
F-statistic	32.26	32.28	32.23	32.26	32.29	32.21	32.31	32.22

References

1. Romer CD, Romer DH. The macroeconomic effects of tax changes: Estimates based on a new measure of fiscal shocks. Am Econ Rev. 2010;100: 763–801. doi:10.1257/aer.100.3.763
2. Hausman JA. Specification Tests in Econometrics. Econometrica. 1978;46: 1251–1271.
3. Furceri D, Ganslmeier M, Ostry JD, Yang N. Initial Output Losses from the Covid-19 Pandemic : Robust Determinants. IMF Work Pap. 2021.
4. Deb P, Furceri D, Ostry JD, Tawk N. The economic effects of COVID-19 containment measures. Covid Econ Vetted Real-Time Pap. 2020;24: 32–35.
5. Zheng B, Geng G, Ciais P, Davis SJ, Martin R V., Meng J, et al. Satellite-based estimates of decline and rebound in China's CO2 emissions during COVID-19 pandemic. Sci Adv. 2020;6. doi:10.1126/sciadv.abd4998
6. Santamaria C, Sermi F, Spyratos S, Iacus SM, Annunziato A, Tarchi D, et al. Measuring the impact of COVID-19 confinement measures on human mobility using mobile positioning data. A European regional analysis. Saf Sci. 2020;132: 104925. doi:10.1016/j.ssci.2020.104925
7. Cerdeiro DA, Komaromi A. Supply Spillovers During the Pandemic : Evidence from High-Frequency Shipping Data. IMF Work Pap. 2020.
8. Petherick A, Hale T, Phillips T, Webster S. Variation in government responses to COVID-19 | Blavatnik School of Government. 2020. Available: https://www.bsg.ox.ac.uk/research/publications/variation-government-responses-covid-19
9. Hummels D, Ishii J, Yi KM. The nature and growth of vertical specialization in world trade. J Int Econ. 2001;54: 75–96. doi:10.1016/S0022-1996(00)00093-3
10. Lenzen M, Kanemoto K, Moran D, Geschke A. Mapping the structure of the world economy. Environ Sci Technol. 2012;46: 8374–8381. doi:10.1021/es300171x
11. Verschuur J, Koks EE, Hall JW. Observed impacts of the COVID-19 pandemic on global trade. Nat Hum Behav. 2021; 1–3. doi:10.1038/s41562-021-01060-5
12. Gaulier G, Zignago S. BACI : International Trade Database at the Product-level The 1994-2007 Version. 2010.
13. Hsiang S, Allen D, Annan-Phan S, Bell K, Bolliger I, Chong T, et al. The effect of large-scale anti-contagion policies on the COVID-19 pandemic. Nature. 2020. doi:10.1038/s41586-020-2404-8
