# Supplementary material for: Global economic impacts of COVID-19 lockdown measures stand out in high-frequency shipping data
Source: PLoS One. 2021 Apr 14;16(4):e0248818. doi: 10.1371/journal.pone.0248818 (PMC8046185; doi:10.1371/journal.pone.0248818)
Supplement: S1 Table — The total trade, imports and exports losses expressed in million tonnes (MT). The losses cover the period Jan-Aug 2020 compared to Jan-Aug 2019. (PDF) [file pone.0248818.s003.pdf]

S1 Table. The top 20 largest negative maritime trade losses on a country-level. The total trade losses and imports and export losses expressed in million tonnes (MT). The losses cover the period Jan-Aug 2020 compared to Jan-Aug 2019.

	Total trade		Imports		Exports	
Rank	Country	Change (MT)	Country	Change (MT)	Country	Change (MT)
1	China	-342.4	China	-217.8	China	-124.6
2	Saudi Arabia	-98.3	United States	-32.4	Saudi Arabia	-99.1
3	United States	-91.3	Netherlands	-28.6	Australia	-60.7
4	Australia	-65.1	Japan	-17.7	United States	-58.9
5	United Arab Emirates	-47.3	India	-14.2	Brazil	-39.5
6	Netherlands	-46.2	United Arab Emirates	-9.8	United Arab Emirates	-37.6
7	Brazil	-44.2	Italy	-8.8	Japan	-18.8
8	Japan	-36.5	Mexico	-8.5	Russia	-18.7
9	Canada	-21.9	Great Britain	-8.3	Netherlands	-17.6
10	Russia	-20.3	Belgium	-7.2	Columbia	-16.2
11	Columbia	-17.1	Kuwait	-7.1	Canada	-15.5
12	Peru	-16.9	Canada	-6.3	Peru	-13.0
13	Italy	-16.4	Brazil	-4.7	Malaysia	-9.7
14	Malaysia	-13.0	South-Korea	-4.6	South-Africa	-8.1
15	South-Korea	-12.5	Australia	-4.4	South-Korea	-8.0
16	Belgium	-11.9	France	-4.3	Libya	-7.9
17	Mexico	-11.9	Iran	-4.0	Italy	-7.7
18	Kuwait	-10.8	Peru	-3.8	Ukraine	-6.9
19	Great Britain	-9.1	Egypt	-3.8	Singapore	-6.5
20	Thailand	-8.6	Thailand	-3.5	Indonesia	-6.0
