# Supplementary material for: Global economic impacts of COVID-19 lockdown measures stand out in high-frequency shipping data
Source: PLoS One. 2021 Apr 14;16(4):e0248818. doi: 10.1371/journal.pone.0248818 (PMC8046185; doi:10.1371/journal.pone.0248818)
Supplement: Attachment — Submitted filename: PONE-D-20-33893_reviewer RV.pdf [file pone.0248818.s004.pdf]

PLOS ONE

The implications of large-scale containments policies on global maritime trade during the COVID-19 pandemic

--Manuscript Draft--

**Manuscript Number:**	PONE-D-20-33893
**Article Type:**	Research Article
**Full Title:**	The implications of large-scale containments policies on global maritime trade during the COVID-19 pandemic
**Short Title:**	The implications of large-scale containments policies on global maritime trade during the COVID-19 pandemic
**Corresponding Author:**	Jasper Verschuur<br>University of Oxford<br>Oxford, UNITED KINGDOM
**Keywords:**	COVID19; economic impacts; maritime trade; automatic identification system; containment measures
**Abstract:**	The implementation of large-scale containment measures by governments to contain the spread of the COVID-19 virus has resulted in a large supply and demand shock throughout the global economy. Here, we use empirical vessel tracking data and a newly developed algorithm to estimate the global maritime trade losses during the first eight months of the pandemic. Our results show widespread trade losses on a port level with the largest absolute losses found for ports in China, the Middle-East and Western Europe, associated with the collapse of specific supply-chains (e.g. oil, vehicle manufacturing). In total, we estimate that global maritime trade reduced by -7.0% to -9.6% during the first eight months of 2020, which is equal to around 206-286 million tonnes in volume losses and up to 225-412 billion USD in value losses.The fishery, mining and quarrying, electrical equipment and machinery manufacturing, and transport equipment manufacturing sectors are hit hardest, with losses up to 11.8%.Moreover, we find a large geographical disparity in losses, with some small islands developing states and low-income economies suffering the largest relative trade losses. We find a clear negative impact of COVID-19 related business and public transport closures on country-wide exports. Overall, we show how real-time indicators of economic activity can support governments and international organisations in economic recovery efforts and allocate funds to the hardest hit economies and sectors.
**Order of Authors:**	Jasper Verschuur
	Elco Koks
	Jim Hall
**Additional Information:**	
**Question**	**Response**
**Financial Disclosure**<br><br>Enter a financial disclosure statement that describes the sources of funding for the work included in this submission. Review the submission guidelines for detailed requirements. View published research articles from *PLOS ONE* for specific examples.<br><br>This statement is required for submission and **will appear in the published article** if the submission is accepted. Please make	This research is supported by the University of Oxford COVID-19 Research Response Fund. J.V. acknowledges funding from the Engineering and Physical Sciences Research Council (EPSRC, https://epsrc.ukri.org) under grant number EP/R513295/1.<br><br>The funders had no role in study design, data collection and analysis, decision to publish, or preparation of the manuscript.

Powered by Editorial Manager® and ProduXion Manager® from Aries Systems Corporation

sure it is accurate.<br><br>**Unfunded studies**<br>Enter: *The author(s) received no specific funding for this work.*<br><br>**Funded studies**<br>Enter a statement with the following details:<br>• Initials of the authors who received each award<br>• Grant numbers awarded to each author<br>• The full name of each funder<br>• URL of each funder website<br>• Did the sponsors or funders play any role in the study design, data collection and analysis, decision to publish, or preparation of the manuscript?<br>• **NO** - Include this sentence at the end of your statement: *The funders had no role in study design, data collection and analysis, decision to publish, or preparation of the manuscript.*<br>• **YES** - Specify the role(s) played.<br><br>* typeset	
**Competing Interests**<br><br>Use the instructions below to enter a competing interest statement for this submission. On behalf of all authors, disclose any competing interests that could be perceived to bias this work—acknowledging all financial support and any other relevant financial or non-financial competing interests.<br><br>This statement **will appear in the published article** if the submission is accepted. Please make sure it is accurate. View published research articles from *PLOS ONE* for specific examples.	The authors have declared that no competing interests exist.

Powered by Editorial Manager® and ProduXion Manager® from Aries Systems Corporation

**NO authors have competing interests**<br><br>Enter: *The authors have declared that no competing interests exist.*<br><br>**Authors with competing interests**<br><br>Enter competing interest details beginning with this statement:<br><br>*I have read the journal's policy and the authors of this manuscript have the following competing interests: [insert competing interests here]*<br><br>* typeset	
**Ethics Statement**<br><br>Enter an ethics statement for this submission. This statement is required if the study involved:<br><br>• Human participants<br>• Human specimens or tissue<br>• Vertebrate animals or cephalopods<br>• Vertebrate embryos or tissues<br>• Field research<br><br>Write "N/A" if the submission does not require an ethics statement.<br><br>General guidance is provided below. Consult the submission guidelines for detailed instructions. **Make sure that all information entered here is included in the Methods section of the manuscript.**	N/A

Powered by Editorial Manager® and ProduXion Manager® from Aries Systems Corporation

**Format for specific study types**<br><br>**Human Subject Research (involving human participants and/or tissue)**<br>• Give the name of the institutional review board or ethics committee that approved the study<br>• Include the approval number and/or a statement indicating approval of this research<br>• Indicate the form of consent obtained (written/oral) or the reason that consent was not obtained (e.g. the data were analyzed anonymously)<br><br>**Animal Research (involving vertebrate animals, embryos or tissues)**<br>• Provide the name of the Institutional Animal Care and Use Committee (IACUC) or other relevant ethics board that reviewed the study protocol, and indicate whether they approved this research or granted a formal waiver of ethical approval<br>• Include an approval number if one was obtained<br>• If the study involved *non-human primates*, add *additional details* about animal welfare and steps taken to ameliorate suffering<br>• If anesthesia, euthanasia, or any kind of animal sacrifice is part of the study, include briefly which substances and/or methods were applied<br><br>**Field Research**<br><br>Include the following details if this study involves the collection of plant, animal, or other materials from a natural setting:<br>• Field permit number<br>• Name of the institution or relevant body that granted permission	
**Data Availability**<br><br>Authors are required to make all data underlying the findings described fully available, without restriction, and from the time of publication. PLOS allows rare exceptions to address legal and ethical concerns. See the PLOS Data Policy and FAQ for detailed information.	Yes - all data are fully available without restriction

A Data Availability Statement describing where the data can be found is required at submission. Your answers to this question constitute the Data Availability Statement and **will be published in the article**, if accepted.<br><br>**Important:** Stating 'data available on request from the author' is not sufficient. If your data are only available upon request, select 'No' for the first question and explain your exceptional situation in the text box.<br><br>Do the authors confirm that all data underlying the findings described in their manuscript are fully available without restriction?	
**Describe where the data may be found in full sentences. If you are copying our sample text, replace any instances of XXX with the appropriate details.**<br><br>• If the data are **held or will be held in a public repository**, include URLs, accession numbers or DOIs. If this information will only be available after acceptance, indicate this by ticking the box below. For example: *All XXX files are available from the XXX database (accession number(s) XXX, XXX.*).<br>• If the data are all contained **within the manuscript and/or Supporting Information files**, enter the following: *All relevant data are within the manuscript and its Supporting Information files.*<br>• If neither of these applies but you are able to provide **details of access elsewhere**, with or without limitations, please do so. For example:<br><br>*Data cannot be shared publicly because of [XXX]. Data are available from the XXX Institutional Data Access / Ethics Committee (contact via XXX) for researchers who meet the criteria for access to confidential data.*<br><br>*The data underlying the results presented in the study are available from (include the name of the third party*	All the derived datasets that can reproduce the results can be accessed from Zenodo: 10.5281/zenodo.4146993

and contact information or URL).<br>• This text is appropriate if the data are owned by a third party and authors do not have permission to share the data.<br><br>* typeset	
Additional data availability information:	Tick here if the URLs/accession numbers/DOIs will be available only after acceptance of the manuscript for publication so that we can ensure their inclusion before publication.

Powered by Editorial Manager® and ProduXion Manager® from Aries Systems Corporation

Manuscript Click here to access/download;Manuscript;Manuscript.docx

The implications of large-scale containments policies on global maritime trade during the COVID-19 pandemic

J.Verschuur[1,*], E.E. Koks[1,2] and J.W. Hall[1]

[1]Environmental Change Institute, University of Oxford, Oxford, United Kingdom

[2]Institute for Environmental Studies, Vrije Universiteit Amsterdam, Amsterdam, Netherlands

[*]Corresponding author

Email: jasper.verschuur@keble.ox.ac.uk (JV)

Abstract

The implementation of large-scale containment measures by governments to contain the spread of the COVID-19 virus has resulted in a large supply and demand shock throughout the global economy. Here, we use empirical vessel tracking data and a newly developed algorithm to estimate the global maritime trade losses during the first eight months of the pandemic. Our results show widespread trade losses on a port level with the largest absolute losses found for ports in China, the Middle-East and Western Europe, associated with the collapse of specific supply-chains (e.g. oil, vehicle manufacturing). In total, we estimate that global maritime trade reduced by -7.0% to -9.6% during the first eight months of 2020, which is equal to around 206-286 million tonnes in volume losses and up to 225-412 billion USD in value losses. The fishery, mining and quarrying, electrical equipment and machinery manufacturing, and transport equipment manufacturing sectors are hit hardest, with losses up to 11.8%. Moreover, we find a large geographical disparity in losses, with some small islands developing states and low-income economies suffering the largest relative trade losses. We find a clear negative impact of COVID-19 related business and public transport closures on country-wide exports. Overall, we show how real-time indicators of economic activity can support governments and international organisations in economic recovery efforts and allocate funds to the hardest hit economies and sectors.

Introduction

The emergence and spread of COVID-19, caused by the severe acute respiratory syndrome coronavirus 2 (SARS-CoV-2), has forced countries worldwide to implement large-scale containment measures to reduce the spread of the virus [1–4]. These measures, which include among others international travel restrictions, business closures, prohibition of large-scale private and public gatherings, and mandatory quarantines, have shown to effectively reduce the rate of transmission of the virus [1,3,5]. As a consequence, however, such policies have had large economic repercussions, both in terms of domestic industry output and international trade, due to diminishing production and reduced demand for some products. The resulting demand and supply shocks has further cascaded through complex supply-chains networks, causing spill-over effects to downstream and upstream suppliers, domestically and internationally [6–8].

Previous research has intended to estimate the global macroeconomic impacts of enforcement measures worldwide using model-based approaches, in particular input-output (I-O) analysis and computable general equilibrium (CGE) models [6–9]. For instance, Inoue and Todo [8] estimate that an one month lockdown of Tokyo may lead to a 5.2% contraction of the annual gross domestic production (GDP) of Japan. Lenzen *et al.* [7] estimate the global macroeconomic losses to be around 4.2% of GDP, whereas Guan *et al.* [6] estimate the losses in global value-added to be between 25-40.3%, depending on the modelling scenario adopted. In addition, the World Trade Organisation (WTO) projects that global international trade will contract by 13-32% in 2020 under a number of scenarios of disruptions to economic activity [10]. However, the accuracy of these model-based estimates critically hinge on setting realistic

scenarios of the economic impacts and understanding how supply-chain dynamics disrupt or buffer shocks. This includes making assumptions how enforcement policies impact economies and predicting how countries recover from their economic downturn. Given the large heterogeneity in containment policies implemented, in terms of severity, duration and enforcements [1,11], alongside the complexity of shock propagation through interconnected networks [12,13], the extent to which such model-based approaches can predict the nature and scale of the economic impact currently remains to be seen. Moreover, there is a lack of real-time global observational data to validate and steer model projections, which may cause ill-informed decision-making or misallocation of funds [5]. For instance, trade statistics, published by national statistical agencies, are usually produced with some delay and are more comprehensive for OECD countries. Hence, alternative data sources are needed to provide real-time indicators of economic activity in order to fill existing data gaps and identify trends. For instance, a report by UNCTAD [14] estimated that in the first half of 2020, global port calls have declined by 8.7%, with the largest drop estimated to be in Europe, Australia and Oceania.

Here, we use a novel dataset derived from empirical vessel tracking data in combination with a newly developed algorithm to estimates the changes in international maritime trade flows during the outbreak of the COVID-19 pandemic. Maritime trade flows cover around 80% of the world's trade in terms of volume [15], and can hence be used as a first-order indicator of the status of economic activity in a country. The newly derived dataset has a high spatial (166 countries) and temporal (daily) resolution, which helps to track the impacts of the COVID-19 outbreak the global economy, and the geographical disparity of impacts. To assess the impacts, we first

quantify the total trade losses and gains for ports and countries for the period January-August by comparing the 2020 data to the same period in 2019. Second, we provide a sector-level disaggregation to show how certain sectors have been disproportionally affected by supply and demand changes. Third, we use a panel regression model with fixed effects to estimate the impacts of specific containment policies on exports by making use of the heterogeneity in the diversity, timing and severity of containment measures across countries. Overall, we provide evidence how the impacts to global trade are complex and dependent on the trade-dependencies, sector-composition and policies implemented. The results provide timely information about which economies are hit hardest, thereby helping decision-makers effectively target and prioritize international aid and economic stimulus.

Method

Data and trade estimation

We derive estimates of port-level trade flows (imports and exports) for 1153 ports across 166 countries worldwide using the geospatial location and attributes of maritime vessels (from January 2019 - August 2020). To do this, we use Automatic Identification System (AIS) data, which provides detailed data on the location, speed, direction and vessel characteristics of all trade-carrying vessels with an AIS transponder (that send information to terrestrial or satellite receivers every few seconds-minutes) [16]. This data was obtained through a partnership with the UN Global Platform AIS Task Team initiative (https://unstats.un.org/wiki/display/AIS/), which aims to develop algorithms and methodologies to make AIS data useful for a variety of fields and applications (traffic, economic trade, fisheries, CO_2 emissions).

We develop an algorithm (S1 Appendix) that estimates the trade flows based on the ingoing and outgoing movements of maritime vessels (~3.2 million port calls across 100,000 unique vessels) and their characteristics (e.g. dimensions, utilisation rate, vessel type), going on to disaggregate these trade flows into specific sectors (11 sector classification adopted here). We end up with daily sector-specific trade flow estimates on a port-level, which we aggregate to a country-scale to perform the country-wide impact analysis. This new algorithm significantly advances previous work [17–19] by providing a global scale analysis and being able to provide a sector decomposition.

We validate the results (S1 Appendix) by comparing the derived trade estimates to detailed port-level trade data obtained for five countries (Japan, United Kingdom, United States, New Zealand, Brazil). Moreover, we compare our estimates to country-wide maritime trade flows obtained from UN Comtrade [20] mode of transport data for 27 countries.

Econometric model

The variation in trade losses among countries are driven by the differences in containment measures introduced by countries (in terms of timing, duration, and severity) [5], and due to supply shortages to domestic supply-chains and demand reductions in trade-dependent economies [6]. Large-scale containment measures can directly negatively influence industry output by affecting business operations (e.g. workplace closure, mobility restrictions), or indirectly positively affect industry output through effectively containing the virus outbreak and thereby allowing industrial production processes and transportation of goods to continue. To study the implications of large-scale containment measures on exports (which we use as a proxy of industrial output), we match our daily, country-wide, estimates with data from the

Oxford COVID-19 Government Response Tracker (OxCGRT) [21]. Within OxCGRT, data is collected on the implementation and stringency of containment measures across 160 countries. We utilise reduced-form econometric techniques [22] to estimate the effect of different containment policies on exports across a balanced sample of 122 countries. We express export change as the percentage change in detrended exports in 2020 compared to 2019 (S2 Appendix), which therefore controls for potential seasonality and trends in the data. The time series is first smoothed using a 10-day moving average in order to remove the daily noise and better capture the underlying signal. We further control (see S2 Appendix for discussion) for several factors on a daily-scale, including the number of confirmed cases as a fraction of the population, reduction in demand in trade-dependent countries, the potential reduction in exports due to supply-shortages (through imports that are used for exports, see Hummels et al. [23]), and other endogenous factors that are likely to be serially correlated with exports (which we control for by adding a lag of the export change).

From the OxCGRT [21], we obtained information on nine measures that potentially affect business operations: C1 - School closing; C2 - Workplace closing; C3 - cancel public events; C4 - Restrictions on gatherings; C5 - Close public transport; C6 - Stay at home; C7 - Restrictions on internal movement; C8 - International travel controls; H2 - Testing policy. We scale the severity of the policies on a scale between 0 and 1, thereby assuming a linear relationship between maritime exports and the severity of policies. Moreover, we create a composite stringency index (Stringency) of all policies (C1-C8) by adding all individual policies together and rescaling the index between 0 and 1. We performed multiple robustness checks to evaluate the influence of different model specifications (see S2 Appendix).

Results

Model validation

We find a good fit between the values predicted by our algorithm and the reported trade flows on a port-level (correlation coefficient between 0.52-0.96) and a country-level (correlation coefficient between 0.79-0.98), with a general overestimation for smaller ports, and ports and countries with large trade imbalances (e.g. small islands). For the external validation data, we find correlation coefficients of 0.84-0.86 for the aggregated trade data and 0.73-0.73 for the sector-specific trade data (on a country level). Again, smaller trade flows are harder to predict. The accuracy of the method is also found to be dependent on the coverage of information in the AIS data (some attributes are manually put in), especially information on the vessel draft, which is less frequently reported in developing countries.

Port-level trade flows

In the first eight months of 2020, the number of port calls across all ports reduced by 4.4% compared to same months in 2019. Fig 1a shows the average change in total trade (imports + exports) in terms of volume (in million tonnes, MT) over the months January-August. The vast majority of ports have experienced a decline in total trade, although a number of ports in Brazil, the Gulf of Mexico region, the Middle-East, Australia, and parts of South-Korea and the Philippines have seen an increase in trade in 2020 relative to 2019. The top 20 port with the largest changes in volume in terms of total trade, imports and exports are included in Table 1. The ports with the largest absolute changes in volume are the ports of Ningbo (China, -68.5 MT), Rotterdam (Netherlands, -43.2 MT), Shanghai (China, -32.5 MT), Wuhan (China, -21.6 MT) and

Tubarao (Brazil, -20.7 MT). The largest changes in imports are found for the ports of Ningbo (China, -43.5 MT), Rotterdam (Netherlands, -40.1 MT), Shanghai (-22.4 MT), Zhoushan (China, -22.4 MT) and Amsterdam (Netherlands, -12.2 MT). These ports, and the other ports in the list, function as major gateway ports for a country to import final products (New York-New Jersey, Rotterdam), or are essential for specific supply-chains, such as textiles and electronics manufacturing (Shanghai, Ningbo, Zhoushan), steel and paper manufacturing (Ghent, Amsterdam, Rizhou), car manufacturing (Yokohama) and raw materials (coal imports for Krishnapatnam). The largest export changes are found for the ports of Ningbo (China, -25.0 MT), Tubarao (Brazil, -17.1 MT), Novorossiysk (Russia, -11.5 MT), Wuhan (China, -10.8 MT), Beaumont (USA, -10.6 MT) and Dampier (Australia, -10.2 MT). These ports, and the other top 20 ports with largest export losses, are all important export ports for global supply-chains, including the exports of iron ore (Dampier, Tubarao), coal (Haypoint), oil and refined petroleum products (Puerto Bolivar, Fujairah, Beaumont and Novorossiysk) and manufacturing products (Ningbo, Wuhan and Shanghai).

Fig 1b-m show the changes in total trade per month for all ports and the cumulative changes in trade over latitude and longitude. In January, losses are predominantly pronounced in China that extended their Lunar New Year holiday [24], among other measures, resulting in output losses to the Chinese industry. This resulted in a direct demand shock, in particular for the export of raw materials (e.g. iron ore, copper, nickel) that China predominantly imports [25]. This can be observed from the large negative losses found in the large export ports of Brazil. In February, ports in Europe experienced their first drop in imports (blue line top plot), while export losses are still concentrated in Asia. This import drop in Europe coincided with the transit time from China to Europe, which is around three weeks. The export drop is, alongside Brazil,

also visible in the main iron ore exporting ports in Australia (Port Hedland and Port Walcott) and South Africa (Port of Richards Bay) that both supply iron ore to the Chinese industry. In March, exports temporally recovered, while imports dropped in many parts of the world, mainly to due to initiation of lockdowns in economies outside Asia. In particular, India, Malaysia, Singapore, USA West Coast and Mexico have seen a large drop in trade in this month. In April, trade partly recovers in the Northern Hemisphere, while in May the second drop in global trade hits the global economy, as a widespread reduction in demand and supply ripple through the economy. Losses are again pronounced in China and Western Europe, leading to the lowest total import and exports changes on a global scale. In June, July and August, a partial recovery is visible for some ports, while the Middle-East, Eastern Australia, Japan and Western Europe (in particular Belgium and the Netherlands) show large losses. For the Middle-Eastern countries, the collapse of the oil market has contributed to the large trade losses (which are predominantly exports losses). In August, signs of recovery (especially imports) are visible for the Philippines, India, South Africa, Brazil and Argentina, and parts of the Mediterranean, while other countries are still experiencing large losses.

Fig 1. Port-level trade losses over time. The geographical location and magnitude of trade losses for Jan-Aug 2020 compared to 2019, including the average over the eight months and the losses per month. Green = positive change, red = negative change. The subplots show the cumulative change over latitude and longitude for imports (dark blue) and exports (dark red).

Table 1. Largest absolute trade losses on a port-level. The top 20 total trade, imports and export losses on a port-level expressed in million tonnes (MT). The losses cover the period Jan-Aug 2020 compared to Jan-Aug 2019.

	**Total trade**			**Imports**			**Exports**		
Rank	Port	iso3	Change (MT)	Port	iso3	Change (MT)	Port	iso3	Change (MT)
1	Ningbo	CHN	-68.5	Ningbo	CHN	-43.5	Ningbo	CHN	-25.0
2	Rotterdam	NLD	-43.2	Rotterdam	NLD	-40.1	Tubarao	BRA	-17.1
3	Shanghai	CHN	-32.5	Shanghai	CHN	-22.4	Novorossiysk	RUS	-11.5
4	Wuhan	CHN	-21.6	Zhoushan	CHN	-13.8	Wuhan	CHN	-10.9
5	Tubarao	BRA	-20.7	Amsterdam	NLD	-12.2	Beaumont	USA	-10.6
6	Zhoushan	CHN	-18.8	Rizhao	CHN	-11.3	Dampier	AUS	-10.2
7	Amsterdam	NLD	-17.4	Wuhan	CHN	-10.7	Shanghai	CHN	-10.1
8	Shekou	CHN	-14.2	Mina Al Ahmadi	KWT	-9.5	Haypoint	AUS	-9.2
9	Hong Kong	HKG	-12.3	Vlissingen	NLD	-8.5	Lumut	MYS	-7.6
10	Vlissingen	NLD	-12.2	Zhanjiang	CHN	-7.6	Shekou	CHN	-7.5
11	Singapore	SGP	-12.1	Umm Said	QAT	-7.4	Tianjin	CHN	-7.3
12	Rizhao	CHN	-11.7	Yokohama	JPN	-7.3	Fujairah	ARE	-7.2
13	Novorossiysk	RUS	-11.7	Ghent	BEL	-7.1	Tangshan	CHN	-6.3
14	Lumut	MYS	-11.6	Singapore	SGP	-6.8	Xiamen	CHN	-6.1
15	Dampier	AUS	-10.8	Hong Kong	HKG	-6.7	Itaqui	BRA	-5.8
16	Yokohama	JPN	-9.9	Shekou	CHN	-6.7	Bohai Bay	CHN	-5.7
17	Haypoint	AUS	-9.7	Krishnapatnam	IND	-6.7	Puerto Bolivar	COL	-5.7
18	Beaumont	USA	-9.5	Magdalla	IND	-6.6	Hong Kong	HKG	-5.6
19	Ghent	BEL	-9.4	Port of Le Havre	FRA	-6.4	Primorsk	RUS	-5.5
20	Zhanjiang	CHN	-9.1	New York-New Jersey	USA	-6.0	Richards Bay	ZAF	-5.4

Geographical disparity

Fig 2a-b show the country-aggregated relative changes in imports and exports, with the top 20 largest negative (relative) changes included in Table 2 and largest negative absolute changes in S1 Table. The top 20 largest total trade losses range between 17-36%. The largest percentage change in imports are associated with small economies such as Turks and Caicos Islands, the Caribbean Netherlands, Bahrain, Anguilla, Federated States of Micronesia and Madagascar (all between 28-37% reduction). Most of the countries with the largest import losses are Small Island Developing States, which are characterised by having large import-dependencies due to their small domestic economies, being reliant on maritime trade flows for trade, and importing large amount of goods to support the tourism sector that constitutes a large share of the country's GDP [26]. With the tourist industry collapsing due to the COVID-19 outbreak [27], the imports are expected to drop further significantly, explaining the widespread reductions observed. Other countries, like a number of countries in Africa, Myanmar, Oman, Philippines, the Baltic States and Sweden have increased their imports, likely due to the increased need for food and medical supplies in developing nations or increased household consumption in some developed countries. In terms of exports, the largest relative losses are found for Libya, New Caledonia, Guinea-Bissau, Northern Mariana Islands, Cape Verde and Sudan (all between 50-78% reduction). These countries include many raw materials exporting countries that have suffered from the demand shock across the world, in particular through trade dependencies with Europe, China and the United States [28]. Moreover, many low income countries had to pro-actively lockdown economies to protect their health care system, or are engaged in economic activities that are less able to be done remotely [28,29]. Some countries

have increased their exports, such as India, Myanmar, Vietnam and Philippines, potentially because of production shifts of manufacturing goods to these countries when factory shut down in China [30]. Moreover, exports grew in Argentina, mainly due to booming exports of food products (e.g. soybeans, beef) to the United States and China [31], and in Tanzania, which increased its exports of gold and food (e.g. nuts) and textile products (e.g. cotton) [32].

Using the World Bank income classification, we test whether high and upper middle income countries have experienced more severe impacts than low and lower middle income countries. Without excluding outliers from the data, we find a significant difference (two-sided t-test with $p>0.05$) between both income groups for exports and imports, with high and upper middle income countries having higher export losses. Hence, although the high and upper middle income countries have higher mean export losses, the most extreme export losses and gains are found for low and lower middle income countries.

Fig 2. Country-level relative trade losses. The relative trade losses for Jan-Aug 2020 compared to 2019 expressed in percentage change. Grey countries indicate no data available.

Table 2. Largest relative trade losses on a country-level. The total trade, imports and export losses on a country-level expressed in million tonnes (MT). The losses cover the period Jan-Aug 2020 compared to Jan-Aug 2019.

	Total trade		Imports		Exports	
Rank	Country	Change (%)	Country	Change (%)	Country	Change (%)
1	Anguilla	-35.6	Turks and Caicos Islands	-36.9	Libya	-77.8
2	Libya	-34.3	Bonaire, Saint Eustatius and Saba	-35.9	New Caledonia	-64.9
3	Federated States of Micronesia	-33.5	Bahrain	-31.3	Guinea-Bissau	-55.6
4	Cape Verde	-30.6	Anguilla	-30.7	Northern Mariana Islands	-54.9
5	Peru	-28.3	Federated States of Micronesia	-29.8	Cape Verde	-53.7
6	Bonaire, Saint Eustatius and Saba	-26.8	Madagascar	-28.4	Sudan	-49.4
7	Malta	-26.2	Timor-Leste	-26.0	Montenegro	-45.1
8	Eritrea	-26.1	Malta	-25.7	Eritrea	-44.6
9	Madagascar	-25.1	Grenada	-22.4	Dem. Republic Congo	-44.3
10	Montenegro	-24.9	Belize	-22.3	Vanuatu	-40.0
11	Turks and Caicos Islands	-24.8	Iran	-21.8	Kenya	-39.6
12	Vanuatu	-24.4	Seychelles	-21.5	Peru	-39.2
13	Seychelles	-23.7	French Polynesia	-21.1	Federated States of Micronesia	-38.4
14	Timor-Leste	-23.3	Aruba	-19.8	American Samoa	-34.9
15	Northern Mariana Islands	-22.2	Vanuatu	-19.4	Albania	-32.6
16	French Polynesia	-21.1	Iraq	-19.3	Seychelles	-28.0
17	Iraq	-20.1	Kuwait	-19.0	Malta	-27.2
18	New Caledonia	-19.3	Macau	-18.3	Yemen	-25.8
19	Bulgaria	-19.1	Bulgaria	-17.4	Romania	-25.6
20	Romania	-17.6	Cape Verde	-17.2	Saint Vincent and the Grenadines	-23.7

Time series of total and sector-specific trade changes

The total trade losses are not uniform across sectors. Fig 3 shows the estimated total trade losses over time (Fig 3a) together with the trade losses for the 11 sector classification considered. The total trade losses are found to be between -7.0% and -9.6% (mean -8.3%), which is equal to around 206-286 MT in volume losses and up to 225-412 billion USD in trade value (uncertainty due to differences in total import and export losses and due to the volume to value conversion). The time series show (Fig 3a) a clear initial drop in trade in the first three months, after which trade partly recovers, followed by a second, more pronounced, drop in trade. In late August 2020, a sign of economic recovery is not yet visible.

Some supply-chains have been more resilient than others. The most resilient sectors are found to be Textiles and wearing apparel (-4.1%), Food and beverages (-5.8%), Other manufacturing (-6.0%) and Wood and paper (-6.3%). The times series of Textiles and wearing apparel and Other manufacturing show, however, a large drop in exports in the early stages of the pandemic, mainly associated with production in China and other Asian economies (e.g. Bangladesh, Malaysia), followed by a gradual recovery and a less steep second drop. The Wood and paper and Food and beverages sectors have been more stable throughout pandemic outbreak, as supply-chains were not significantly disrupted, and demand for products only gradually declined, followed by signs of a recovery at the end of August. The largest relative changes are found for the Fishing sector (-9.5%), Mining and quarrying (-9.0%), Manufacturing of electronics and machinery (-8.8%), and Manufacturing of transport equipment (-11.8%). The drop in fishing products peaked late in the pandemic with a clear recovery in July and August. The time series of the mining and quarrying sector shows a more complex picture with a sharp drop in the beginning of the pandemic, as demand for raw materials decreased

in Asia, followed by a steep increase in trade to restock inventories, after which a total collapse of the market can be observed, mainly associated with reduced demand for oil. For the two manufacturing sectors, the large losses are the result of significant supply-chain disruptions that caused upstream production processes to halt due to a shortage in supplies [8]. In particular the Transport manufacturing industry, characterised by just-in-time logistic services and highly specialised production processes, experienced a gradual disruption throughout the first few months, after which trade declined more than 20% in May, June and July.

Fig 3. Sector-specific losses over time. The change in daily global total trade as a fraction of the average daily trade (over 2019). The dark blue line represent imports, the dark red line represent exports, whereas the grey line indicate total trade (import + exports). Sector 1: Agriculture; Sector 2: Fishing; Sector 3: Mining and quarrying; Sector 4: Food and beverages; Sector 5: Textiles and wearing apparel; Sector 6: Wood and paper; Sector 7: Petroleum, chemical and non-metallic mineral products; Sector 8: Metal products; Sector 9: Electrical and machinery; Sector 10: Transport equipment; Sector 11: Other manufacturing.

Impact of large-scale containment measures

The results of the panel regression model are included in ~~Table 1.~~ After testing various model specifications (S1 Appendix), we find the most robust results for the model that includes daily control variables for the number of confirmed cases as a fraction of the population (Cases), demand reduction in trade dependent countries (Demand), the potential supply disruptions through changes in import that are used for exports

(Supply), and potential other factors that are autocorrelated with the changes in export (Export lag).

The effect of the composite index on daily export change is strong, and statistically significant ($p < 0.01$), with a 10% increase of the index resulting in a -0.45% change in exports (Model 1).

The influence of containment measures on exports is mixed with some measures showing a negative impact while others showing a positive impact (Model 2). Negative impacts are found for school closing (C1, - 2.63%, significant at $p < 0.05$), workplace closure (C2, -4.76, significant at $p < 0.01$) and closing of public transport (C5, -3.59% significant at $p < 0.01$). Surpringly, a positive effect is found for stay at home requirements (C6, +2.74%, significant at $p < 0.10$), restrictions on internal movement (C7, +2.17%, significant at $p < 0.10$). Hence, implementing some policies has enabled countries to continue producing export goods and transport them to their ports, although the signal is generally not strong.

The results are potentially biased because of the fact that containment measures may affect domestic production in a lagged manner. In S2 Appendix, we perform additional robustness tests by introducing a 4/8/12/16 day lag of the containment policies. This increases the negative effect of school closures, which varies between -3.36% and -6.87% depending on the lag (all significant at $p < 0.01$), while it increases the positive effect of international travel bans for lags of 8 – 16 days (between +2.85% and 6.03%, significant at $p < 0.01$).

Additionally, we run a model (Model 3 and 4 that includes daily dummy variables to account for economy-wide factors that affect maritime exports) and a model (Model 5 and 6) which include only the days where the outbreak become significant in a country (which we define as having at least 50 confirmed cases). This coefficient of the

composite index is stable across models (-4.53% versus -4.94% and -4.72%, Model 3 and 5). For the individual containment measures, the effect of school closures is larger in Model 4 (-2.63%, significant at $p < 0.05$) and Model 6 (-6.53%, significant at $p < 0.01$). The positive effect of testing is enhanced in Model 6 (+2.83%, significant at $p < 0.05$), whereas the positive effect of stay at home policies becomes larger and statistically significant in Model 4 and 6 (+2.74%, significant at $p < 0.10$; +6.53%, significant at $p < 0.01$). The results change little when performing additional robust checks by modifying the number of days of the lag of the export and adding week dummies (instead of day dummies) to the model (see S2 Appendix).

Across all model specifications, we find a consistent negative relationship between the overall stringency index and maritime exports, and business and public transport closures and maritime exports, whereas all other policies are either not significant, or not consistently significant across different model specifications.

Table 3. The results of the various regression models. The table shows the estimated beta coefficients and goodness of fit statistics for the six model specifications discussed. *p < 0.1, **p < 0.05, ***p < 0.01

	Model1	Model2	Model3	Model4	Model5	Model6
Parameter	Beta	Beta	Beta	Beta	Beta	Beta
Composite	-4.527***		-4.939***		-4.717***	
C1		-1.969		-2.630**		-6.531***
C2		-4.703***		-4.755***		-3.006*
C3		0.557		0.381		1.298
C4		-0.613		-0.822		-2.114
C5		-3.588***		-3.500***		-5.429***
C6		2.049		2.740*		6.525***
C7		2.173**		2.191*		1.377
C8		1.825		1.995		3.771
H2		0.890		0.728		2.833**
Demand	-0.015	-0.033	-0.016	-0.014	0.005	-0.022
Cases	-0.129**	-0.113*	-0.130*	-0.124*	-0.393***	-0.427***
Supply	0.018***	0.018***	0.018***	0.018***	-0.003	-0.002
Export lag	0.350***	0.349***	0.351***	0.350***	0.402***	0.399***
R2	0.320	0.321	0.329	0.330	0.359	0.361
R2-adjusted	0.316	0.316	0.318	0.319	0.353	0.355
F-statistic	73.67	69.23	29.84	29.20	64.74	61.18

Discussion and conclusion

We present a near-global analysis of maritime trade indicators based on empirical vessel tracking data. We illustrate how the implementation of large-scale containment measures have resulted in large trade losses, and hence domestic output, with a strong geographical and sectoral heterogeneity.

Our estimate of a 4.4% reduction in global ports calls for the first eight months of 2020 is lower than the 8.7% predicted by UNCTAD for the first six months [14]. The main reason for this difference is associated with the inclusion of different vessel types. Whereas we include only the main trade-carrying vessels, the UNCTAD analysis also

included passengers vessels (66% of total port calls), which have seen the largest drop in port calls (-17% for passenger vessels). Moreover, the sector-level trends we found are in line with the sector-level impacts (based observed trade data of China, the European Union and the United States) for the first quarter (Q1) of 2020 as presented in the UNCTAD analysis [14], that stated that in particular the automotive industry (-8%), machinery (-8%), office machinery (-8%) and textiles and apparel (-11%) are particularly hit. Our analysis, which differs by only including maritime trade (instead of total trade) and having a global scale (instead of three countries), found the average losses in Q1 for the textiles and apparel (Sector 5), electrical equipment and machinery manufacturing (Sector 9), transport equipment (Sector 10) and other manufacturing (Sector 11) to be respectively 6.2%, 9.2%, 6.5% and 8.4%. The trade losses we estimate (225-412 billion USD for the first eight months) are considerably lower than reported in the modelling framework of Lenzen et al. [7], who estimated the trade losses for the first five months of 2020 to be 536 billion USD. Again, part of this difference is due to the coverage of countries (they provide a full global analysis) and modes of transport (all modes compared to maritime only). Still, input-output based analysis, as done in Lenzen et al. [7], often fail to consider adaptative behaviour in the global economic system, which can dampen economic impacts [33]. The -8.6% trade losses we found are also lower than the 26.8%, and up to 31%, value-added losses (although not being directly comparable) reported in the global pandemic scenarios of Guan et al. [6]. We expect the percentage change in trade losses to increase going further in 2020, as daily trade losses are above 10% at the end of August. This is in line with projections of the UNCTAD [14] and the WTO [10] that estimate a further contraction of merchandise trade in the second half of 2020.

The results of the econometric model provide an alternative view to the studies that have evaluated the effect of large-scaled containment measures on the spread of the virus [1,2,4,34]. We find clear evidence of the negative impacts of large-scale containment measures on changes in daily exports, with a 10% increase in the overall stringency value resulting in a 0.45%-0.50% decrease in daily maritime exports. In particular, introducing required closing of all public transport and all-but-essential businesses resulted in large maritime export losses of up to 5.4-6.5% of daily export. This is in agreement with Deb et al. [5], who used nitrogen emissions as an indicator of industrial output and found that workplace closures had the largest influence on the drop in emissions. Results should, however, be interpreted with caution, as many factors could potentially influence these causal relationships. For instance, temporal increases in maritime transport during some periods of the pandemic could be driven by the large increase in trade of medical supplies (e.g. PPE) and mode substitution from air to maritime [35], irrespective if policies were imposed during these periods. Therefore, testing alternative economic indicators, such as data on retail sales, mobility, flight, energy consumption and nitrogen emissions, as done in Deb et al. [5] can help support these findings. Overall, our analysis of the economic implications of introducing containment policies into society can help evaluating the cost-benefit of the different containments measures, which may help governments construct effective portfolios of policies as many countries enter a second-wave of COVID-19 cases [36]. Future work can refine our estimates by adding more trade data when it becomes available, including extending the analysis to indicators of air, road and rail transport. Moreover, the empirical estimates derived here can be used to constrain and validate macro-economic impact models in order to improve the quantification of the total losses to industrial output as the pandemic unfolds.

Overall, real-time indicators of economic activity, such as maritime trade, can help identify trends in supply-chains networks and support governments and international organisations in their economic recovery efforts by allocating funds to the hardest hit economies and sectors.

Acknowledgements

All derived datasets used for this analysis are made publicly available at Zenodo: 10.5281/zenodo.4146993 and will be published upon acceptance. The policy indicators are obtained from the Oxford Coronavirus Government Response Tracker (https://www.bsg.ox.ac.uk/research/research-projects/coronavirus-government-response-tracker).

The authors would like to thank the United Nations Statistical Division and the UN Global Working Group on Big Data for Official Statistics, in particular Markie Muryawan and Ronald Jansen, for providing the AIS data.

Supporting information

S1 Appendix: Methodology maritime trade estimates

S2 Appendix: Econometric model

S1 Table. The top 20 largest negative maritime trade losses on a country-level. The total trade, imports and exports losses expressed in million tonnes (MT). The losses cover the period Jan-Aug 2020 compared to Jan-Aug 2019.

References

1. Hsiang S, Allen D, Annan-Phan S, Bell K, Bolliger I, Chong T, et al. The effect of large-scale anti-contagion policies on the COVID-19 pandemic. Nature. 2020. doi:10.1038/s41586-020-2404-8
2. Kraemer MUG, Yang C-H, Gutierrez B, Wu C-H, Klein B, Pigott DM, et al. The effect of human mobility and control measures on the COVID-19 epidemic in China. Science (80-). 2020;21: eabb4218. doi:10.1126/science.abb4218
3. Chinazzi M, Davis JT, Ajelli M, Gioannini C, Litvinova M, Merler S, et al. The effect of travel restrictions on the spread of the 2019 novel coronavirus (COVID-19) outbreak. Science. 2020;9757: 1–12. doi:10.1126/science.aba9757
4. Tian H, Liu Y, Li Y, Wu C-H, Chen B, Kraemer MUG, et al. An investigation of transmission control measures during the first 50 days of the COVID-19 epidemic in China. Science (80-). 2020;368: 638–642. doi:10.1126/science.abb6105
5. Deb P, Furceri D, Ostry JD, Tawk N. The economic effects of COVID-19 containment measures. Covid Econ Vetted Real-Time Pap. 2020;24: 32–35.
6. Guan D, Wang D, Hallegatte S, Davis SJ, Huo J, Li S, et al. Global supply-chain effects of COVID-19 control measures. Nat Hum Behav. 2020. doi:10.1038/s41562-020-0896-8
7. Lenzen M, Li M, Malik A, Pomponi F, Sun Y-Y, Wiedmann T, et al. Global socio-economic losses and environmental gains from the Coronavirus pandemic. PLoS One. 2020;15: e0235654. doi:10.1371/journal.pone.0235654
8. Inoue H, Todo Y. The propagation of economic impacts through supply chains:

The case of a mega-city lockdown to prevent the spread of COVID-19. Xing L, editor. PLoS One. 2020;15: e0239251. doi:10.1371/journal.pone.0239251
9. McKibbin WJ, Fernando R. The Global Macroeconomic Impacts of COVID-19: Seven Scenarios. SSRN Electron J. 2020. doi:10.2139/ssrn.3547729
10. WTO. Methodology for the WTO Trade Forecast of April 8 2020. 2020.
11. Cheng C, Barceló J, Hartnett AS, Kubinec R, Messerschmidt L. COVID-19 Government Response Event Dataset (CoronaNet v.1.0). Nat Hum Behav. 2020;4: 756–768. doi:10.1038/s41562-020-0909-7
12. Henriet F, Hallegatte S, Tabourier L. Firm-network characteristics and economic robustness to natural disasters. J Econ Dyn Control. 2012;36: 150–167. doi:10.1016/j.jedc.2011.10.001
13. Inoue H, Todo Y. Propagation of negative shocks across nationwide firm networks. PLoS One. 2019;14: 1–17. doi:10.1371/journal.pone.0213648
14. UNCTAD. COVID-19 and maritime transport : Impact and responses. Geneva; 2020. Report No.: UNCTAD/DTL/TLB/INF/2020/1.
15. UNCTAD. Review of Maritime Transport 2017 - Developments in International Seaborn Trade. 2017.
16. Verschuur J, Koks EE, Hall JW. Port disruptions due to natural disasters: Insights into port and logistics resilience. Transp Res Part D Transp Environ. 2020;85: 102393. doi:10.1016/j.trd.2020.102393
17. Adland R, Jia H, Strandenes SP. Are AIS-based trade volume estimates reliable? The case of crude oil exports. Marit Policy Manag. 2017;44: 657–665. doi:10.1080/03088839.2017.1309470
18. Arslanalp S, Marini M, Tumbarello P. Big Data on Vessel Traffic: Nowcasting Trade Flows in Real Time. IMF Work Pap. 2019;2019.

doi:10.5089/9781513521121.001

19. Cerdeiro DA, Komaromi A, Liu Y, Saeed M. World Seaborne Trade in Real Time : A Proof of Concept for Building AIS-based Nowcasts from Scratch. 2020. Report No.: WP/20/XX World.

20. United Nations Statistical Division. UN Comtrade database. In: UN Comtrade database [Internet]. 2020 [cited 1 Feb 2020]. Available: https://comtrade.un.org

21. Petherick A, Hale T, Phillips T, Webster S. Variation in government responses to COVID-19 | Blavatnik School of Government. 2020. Available: https://www.bsg.ox.ac.uk/research/publications/variation-government-responses-covid-19

22. Romer CD, Romer DH. The macroeconomic effects of tax changes: Estimates based on a new measure of fiscal shocks. Am Econ Rev. 2010;100: 763–801. doi:10.1257/aer.100.3.763

23. Hummels D, Ishii J, Yi KM. The nature and growth of vertical specialization in world trade. J Int Econ. 2001;54: 75–96. doi:10.1016/S0022-1996(00)00093-3

24. Chen S, Yang J, Yang W, Wang C, Bärnighausen T. COVID-19 control in China during mass population movements at New Year. Lancet. 2020;395: 764–766. doi:10.1016/S0140-6736(20)30421-9

25. Mathews JA, Tan H. Circular economy: Lessons from China. Nature. 2016;531: 440–442. doi:10.1038/531440a

26. Briguglio L. Small island developing states and their economic vulnerabilities. World Dev. 1995;23: 1615–1632. doi:10.1016/0305-750X(95)00065-K

27. Gössling S, Scott D, Hall CM. Pandemics, tourism and global change: a rapid assessment of COVID-19. J Sustain Tour. 2020;0: 1–20. doi:10.1080/09669582.2020.1758708

28. Gondwe G. Assessing the Impact of COVID-19 on Africa’s Economic Development. 2020. Available: https://unctad.org/en/PublicationsLibrary/aldcmisc2020d3_en.pdf

29. Adam C, Henstridge M, Lee S. After the lockdown: macroeconomic adjustment to the COVID-19 pandemic in sub-Saharan Africa. Oxford Rev Econ Policy. 2020. doi:10.1093/oxrep/graa023

30. Nikkei Asian Review. Google, Microsoft shift production from China faster due to virus. In: Coronavirus [Internet]. 2020 [cited 1 Jul 2020]. Available: https://asia.nikkei.com/Spotlight/Coronavirus/Google-Microsoft-shift-production-from-China-faster-due-to-virus

31. Buenos Aires Times. China overtook Brazil as Argentina’s largest trading partner in April. In: Economy [Internet]. 2020 [cited 1 Sep 2020]. Available: https://www.batimes.com.ar/news/economy/china-overtook-brazil-as-argentinas-largest-trading-partner-in-april.phtml

32. Tanzania Invest. Tanzania Exports Up 15% in April 2020. In: Tanzania Trade [Internet]. 2020 [cited 1 Oct 2020]. Available: https://www.tanzaniainvest.com/economy/trade/exports-april-2020

33. Koks EE, Thissen M. A Multiregional Impact Assessment Model for disaster analysis. Econ Syst Res. 2016;28: 429–449. doi:10.1080/09535314.2016.1232701

34. Giordano G, Blanchini F, Bruno R, Colaneri P, Di Filippo A, Di Matteo A, et al. Modelling the COVID-19 epidemic and implementation of population-wide interventions in Italy. Nature Medicine. Springer US; 2020. doi:10.1038/s41591-020-0883-7

35. Freightwaves. More air cargo finds its sea legs during COVID-19. 2020 [cited 7

Oct 2020]. Available: https://www.freightwaves.com/news/more-air-cargo-finds-its-sea-legs-during-covid-19

36. Cacciapaglia G, Cot C, Sannino F. Second wave COVID-19 pandemics in Europe: A Temporal Playbook. Sci Rep. 2020; 1–8. doi:10.1038/s41598-020-72611-5

Fig1

Click here to access/download;Figure;Fig1.tiff

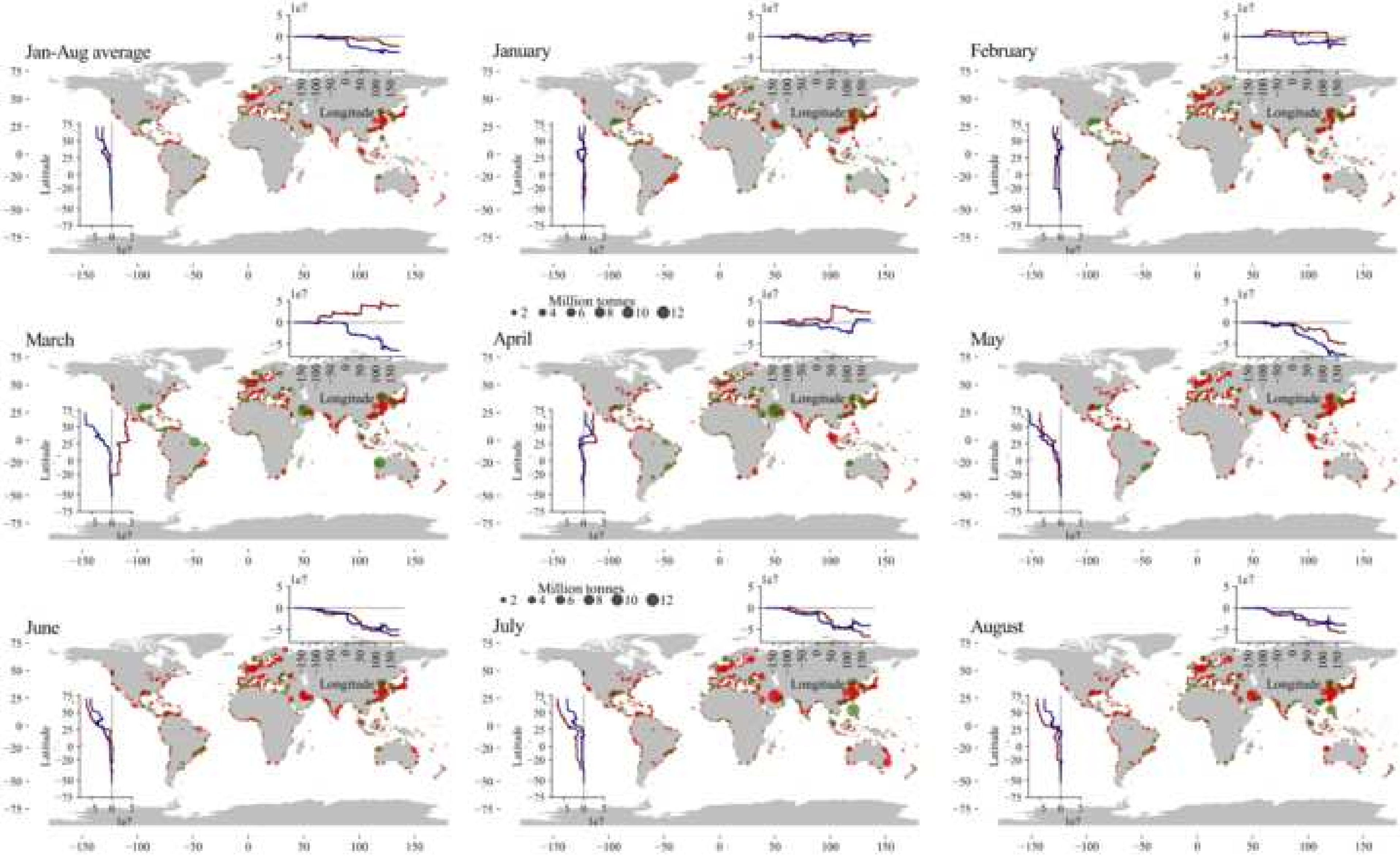

Fig2

Click here to access/download;Figure;Fig2.tiff

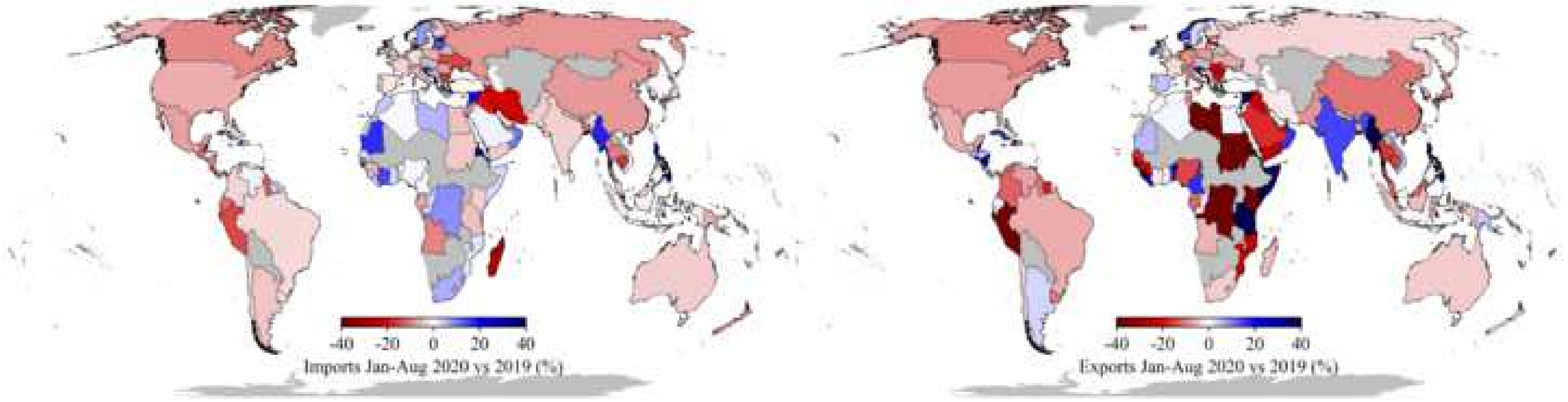

Fig3

Click here to access/download;Figure;Fig3.tiff

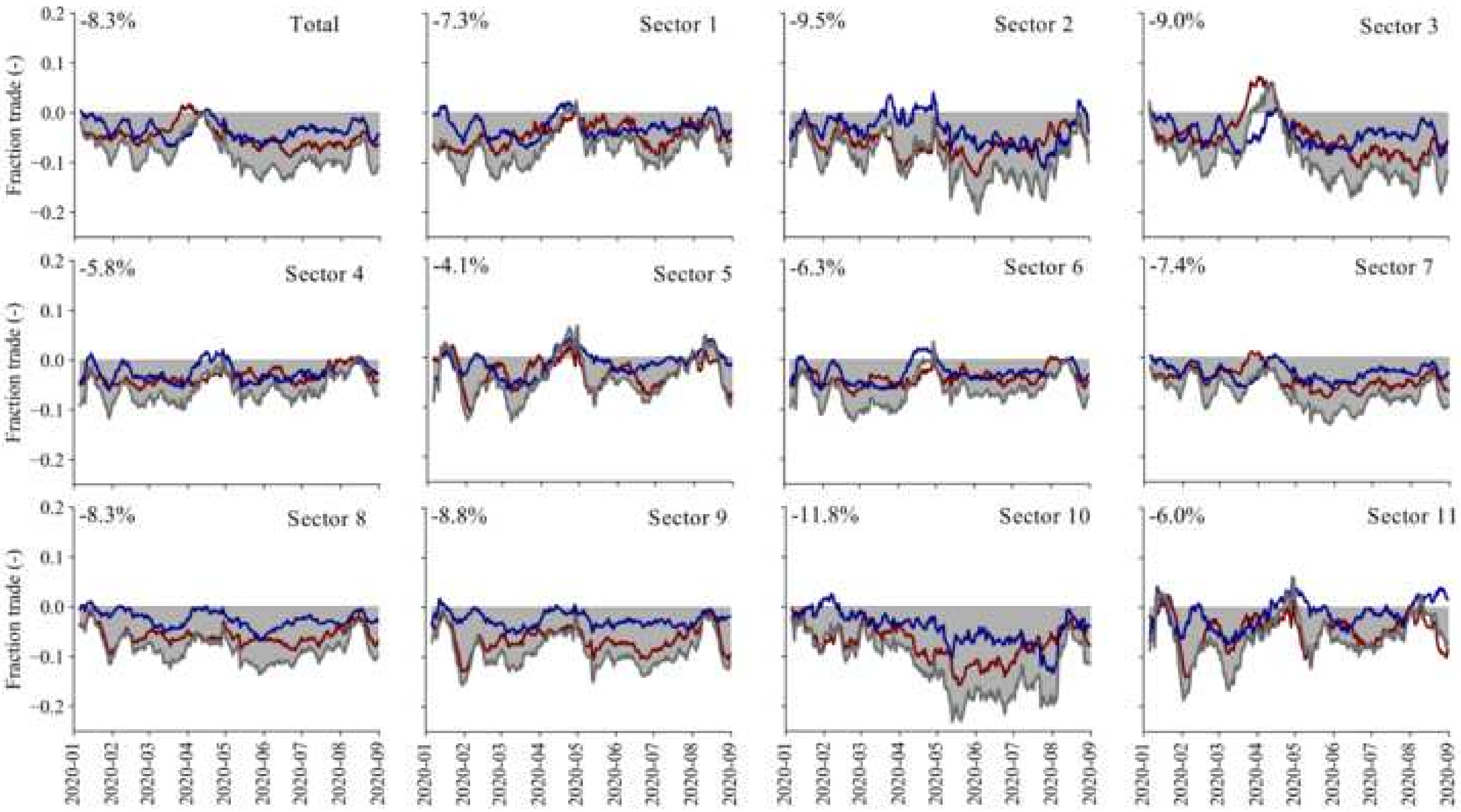

S1 Appendix

Click here to access/download
Supporting Information
S1_appendix.pdf

S2 Appendix

Click here to access/download
Supporting Information
S2_Appendix.pdf

S1 Table

Click here to access/download
Supporting Information
S1_Table.docx
